# Solid-State NMR Reveals Asymmetric ATP Hydrolysis in the Multidrug ABC Transporter BmrA

**DOI:** 10.1021/jacs.2c04287

**Published:** 2022-07-01

**Authors:** Denis Lacabanne, Thomas Wiegand, Margot Di Cesare, Cédric Orelle, Matthias Ernst, Jean-Michel Jault, Beat H. Meier, Anja Böckmann

**Affiliations:** † Physical Chemistry, ETH Zurich, 8093 Zurich, Switzerland; ‡ Molecular Microbiology and Structural Biochemistry, UMR5086 CNRS/University of Lyon, 7, passage du Vercors, 69367 Lyon, France

## Abstract

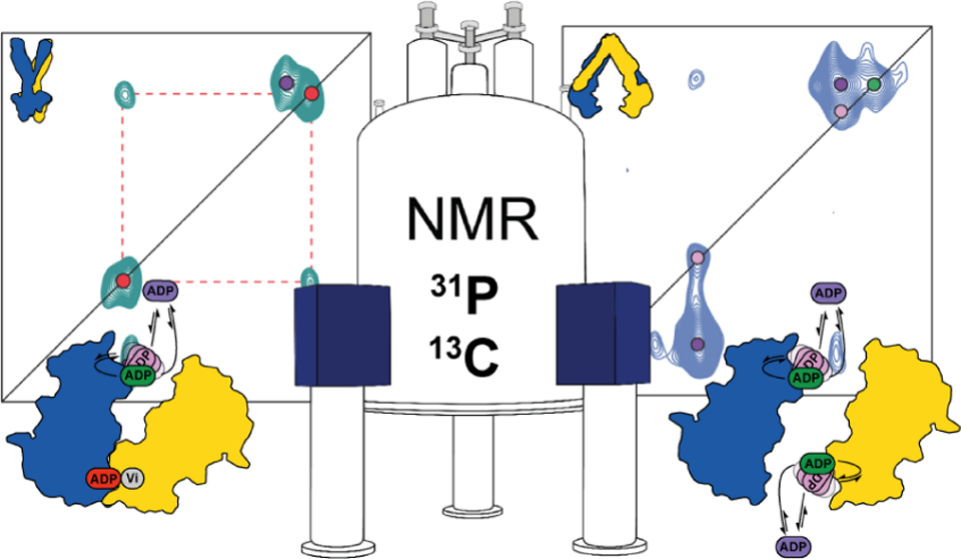

The detailed mechanism of ATP hydrolysis in ATP-binding cassette (ABC) transporters is still not fully understood. Here, we employed ^31^P solid-state NMR to probe the conformational changes and dynamics during the catalytic cycle by locking the multidrug ABC transporter BmrA in prehydrolytic, transition, and posthydrolytic states, using a combination of mutants and ATP analogues. The ^31^P spectra reveal that ATP binds strongly in the prehydrolytic state to both ATP-binding sites as inferred from the analysis of the nonhydrolytic E504A mutant. In the transition state of wild-type BmrA, the symmetry of the dimer is broken and only a single site is tightly bound to ADP:Mg^2+^:vanadate, while the second site is more ‘open’ allowing exchange with the nucleotides in the solvent. In the posthydrolytic state, weak binding, as characterized by chemical exchange with free ADP and by asymmetric ^31^P–^31^P two-dimensional (2D) correlation spectra, is observed for both sites. Revisiting the ^13^C spectra in light of these findings confirms the conformational nonequivalence of the two nucleotide-binding sites in the transition state. Our results show that following ATP binding, the symmetry of the ATP-binding sites of BmrA is lost in the ATP-hydrolysis step, but is then recovered in the posthydrolytic ADP-bound state.

## Introduction

ATP-binding cassette (ABC) transporters are membrane proteins that translocate various molecules across cellular membranes using ATP as an energy source.^1^ They are widespread and can be found in the three kingdoms of life with a remarkable conservation of their ATP-binding motifs.^2^ Their primary function is to mediate the uptake of nutrients in the cells, such as sugars and vitamins, as well as the efflux of a large variety of compounds, and to perform some mechanotransmission tasks.^3^ Some exporters play a major role in detoxification by expelling xenobiotic compounds out of the cell,^4−6^ thereby leading to multidrug resistance phenotypes notably in human anticancer therapies^4,7^ or in pathogenic microorganisms.^6^

**Figure 1:**
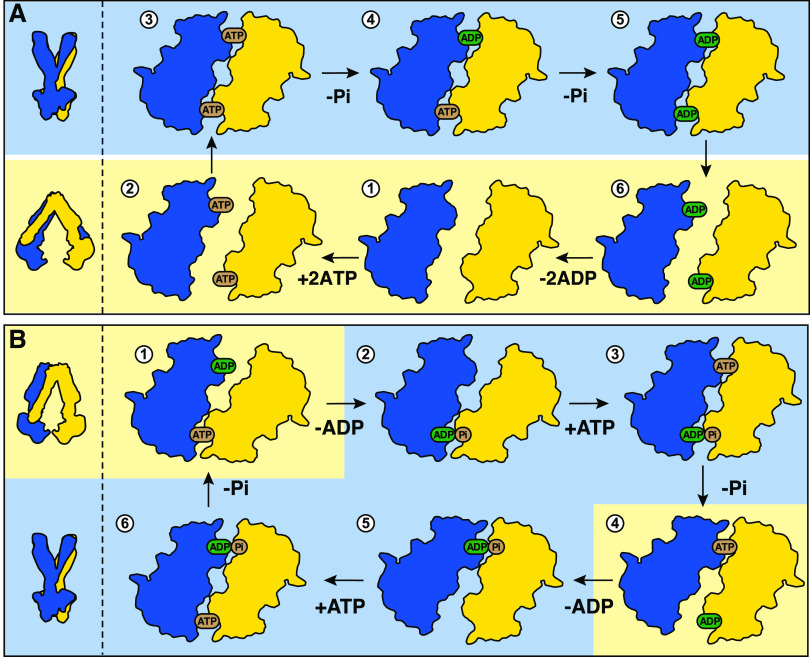
Schematic representation of the (A) ATP switch model or processive clamp model (sequential ATP hydrolysis)^22^ and the (B) constant contact model or alternating site model.^23,9^ In the blue background, the conformation of the transporter is presumably in an outward-facing conformation, and in the yellow background, it is in a putative inward-facing conformation.

ABC transporters contain two nucleotide-binding domains (NBDs) that are able to bind and hydrolyze ATP to harness the chemical energy required for the transport. ATP interacts with several well-conserved motifs and/or residues of the NBDs. During the catalytic cycle, the NBDs engage in a transient tight interaction where two ATP molecules are sandwiched between different motifs, notably the so-called Walker A and B motifs from one NBD and the ABC signature motif from the other NBD.^8^ While the NBDs are highly conserved, the transmembrane domains (TMDs) of ABC transporters are notably divergent in primary sequences and structures. These large differences, and the fact that some ABC transporters contain only one highly active NBD, suggest that a unified model of the ATP-hydrolysis cycle is difficult to establish and a single model might not explain the function of all ABC transporters.^9^ How exactly ATP binding and hydrolysis occur is not fully understood, and two main models were developed: the ATP switch model^10^ (or the related processive clamp model^11^) and the constant contact model^12^ (see Figure 1 for a schematic representation).

In the ATP switch model (Figure 1A), there is a complete separation of the NBDs in the resting state (Figure 1A state **1**, the protein adopts the inward-facing state, yellow background) until the binding of two ATP molecules (step **2**–**3**) promotes the dimerization of the NBDs (to generate the outward-facing state, blue background, Figure 1A, state **3**).^13^ Subsequently, the sequential ATP hydrolysis (steps **3**–**4** and **4**–**5**) induces the dissociation of the NBDs (step **5**–**6**).^14^ This model was mostly derived from static structures of ABC transporters in different states.^10,15^ Its main feature is that drug efflux is directly coupled to the NBD dimerization upon ATP binding, i.e., the transition to the outward-facing state, while ATP hydrolysis will be used to reset the transporter for the next cycle.

In contrast, the constant contact model assumes that each catalytic site contains two different nucleotides (ADP poorly bound and ATP strongly bound) and, as a result, the NBDs would always be in asymmetric states: one site is closed, while the other one is open (Figure 1B). The NBDs always remain in contact throughout the cycle, with opening and closing of the sites occurring via intrasubunit conformational changes between the RecA-like and α-helical subdomains within each NBD monomer. As depicted in Figure 1B, initially two nucleotides are bound to the transporter, an ATP and an ADP. The latter is released, while the ABC transporter adopts the inward-facing conformation (step **1**–**2**, yellow background, Figure 1B). The ABC transporter switches into the outward-facing conformation (blue background, Figure 1B) and the remaining ATP molecule is hydrolyzed into ADP and Pi (step **1**–**2**). ATP binding to the empty site promotes the opening of the other site, switching the transporter into the inward-facing state inducing the release of the Pi and subsequently the ADP molecule (steps **2**–**3**, **3**–**4**, and **4**–**5**). The release of the ADP leads to an empty site and an ATP-bound site, which is hydrolyzed, and then the cycle continues with the opposite ATP-binding site (steps **4**–**5** and **5**–**6**). This model was developed to take into account the asymmetric occupancy of the nucleotide-binding sites observed in structural^16^ or biochemical studies.^17^ Indeed, occlusion of one site during the transition state (in the presence of vanadate) has been observed for several ABC transporters (P-glycoprotein,^18^ LmrA,^19^ or the maltose transporter^20^). This NBD asymmetry in the functioning mechanism also led to alternative models where ATP hydrolysis could be directly coupled to drug efflux.^21^

Isolated NBDs of ABC transporters^24^ have been studied by solution-state nuclear magnetic resonance (NMR). However, solution-state NMR experiments remain a challenge because of the large size of many ABC transporters preventing thus far the spectral attribution of all chemical shifts. By reconstituting ABC transporters in a lipid membrane, solid-state NMR experiments (which do not suffer from size limitations) have been successfully performed on some ABC transporters,^25^ including BmrA.^26,27^ Here, we investigated the different states in the ATP-hydrolysis cycle of BmrA, an ABC transporter from *Bacillus subtilis* involved in antibiotic resistance.^28^ In particular, we analyzed, at a molecular level, the nucleotide-binding modes via ^31^P solid-state NMR of the protein reconstituted in lipids. We also assessed the different nucleotide-bound states by nano-differential-scanning fluorimetry. Our results revealed an asymmetric binding of the two nucleotides in the transition state of ATP hydrolysis, as mimicked by ADP in the presence of vanadate. In contrast, the pre- and posthydrolytic states are symmetric. Our data thus point to a model in which the two NBDs behave in a symmetric mode during the pre- and posthydrolytic steps, while this symmetry is transiently lost during the transition step. Taken together, our results indicate a functioning mechanism of BmrA in agreement with the ATP switch (or processive clamp) model for the pre- and posthydrolytic states, while the transition state resembles more the assumptions of the constant contact model (or the alternating site model). We thus herein propose a new ATP-hydrolysis model for BmrA.

## Materials and Methods

### Production, Purification, and Reconstitution of BmrA

#### Production

The minimal M9-medium used for the bacteria culture was composed of 38 mM anhydrous Na_2_HPO_4_, 8.6 mM NaCl, 22 mM anhydrous KH_2_PO_4_, 2 mM MgSO_4_, 100 μM CaCl_2_, 50 μg·mL^–1^ ampicilline (Sigma-Aldrich A9518), 2 g·L^–1^d-[U-^13^C]glucose (99%) (Cambridge Isotope Laboratories, Inc. CLM-1396-PK), 2 g·L^–1 15^NH_4_Cl (98%) (Sigma-Aldrich 299251), and trace element solution composed of 0.17 mM EDTA, 0.027 mM CuSO_4_, 0.095 mM MnCl_2_, 0.003 mM H_3_BO_3_, 0.024 mM ZnSO_4_, 0.216 mM FeSO_4_, and 0.011 mM ascorbic acid with vitamin cocktail (Sigma-Aldrich B6891).

Selective unlabeling^27−29^ was used for the expression of the protein. Natural abundance amino acids added 1 h prior to the induction are 0.25 g·L^–1^ Ile, 0.25 g·L^–1^ Leu, 0.25 g·L^–1^ Val, 0.40 g·L^–1^ Lys, 0.10 g·L^–1^ Pro, and 0.40 g·L^–1^ His.

For the production of the proteins BmrA and BmrA-E504A, bacteria carrying the pET23b(+)-bmrA or pET23b(+)-bmrA-E504A vector were used. The proteins were expressed using *E. coli* strain C41(DE3), which display a high expression level.^30^ A clone was inoculated into 3 mL of LB medium and incubated for 4 h at 37 °C and 200 rpm (preculture 1). Then, 50 mL of minimal M9-medium (preculture 2) in a baffled flask (150 mL) was inoculated with 3 mL of the preculture 1 and incubated at 37 °C and 200 rpm until an OD_600 nm_ of 1.5. A third preculture (preculture 3) of 150 mL minimal M9-medium in baffled flasks (500 mL) was inoculated with the preculture 2 to an initial OD_600 nm_ of 0.2 and incubated overnight at 25 °C and 130 rpm. Finally, four 2L baffled flasks containing 425 mL of minimal M9-medium were inoculated with 75 mL of the preculture 3 and incubated at 25 °C and 130 rpm. The expression of BmrA was induced with 0.7 mM IPTG when the OD_600 nm_ reached 0.6–0.7. The cultures were incubated until the stationary phase was reached. The bacteria were harvested by centrifugation at 6000*g* during 20 min at 4 °C. Bacterial lysis was performed using a high-pressure homogenizer Microfluidizer and was followed by 15 000*g* (4 °C for 15 min) and 200 000*g* (4 °C for 1 h) centrifugation steps to harvest the membrane containing the overexpressed BmrA.

#### Purification

The bacterial membranes were diluted at 2 mg.mL^–1^ with a solubilization buffer (50 mM Tris-HCl pH 8, 100 mM NaCl, 1% n-dodecyl-β-d-maltopyranoside (DDM), 1 mM DTT, 15% glycerol) and incubated for 1 h at 4 °C under rotation. The solubilized protein was incubated in a batch with Ni^2+^-nitrilotriacetic acid- (Ni-NTA) agarose resin column (Qiagen) equilibrated with 5 column volumes (CV) of equilibrating buffer (EB) (50 mM Tris-HCl, pH 8, 100 mM NaCl, 15% glycerol, 0.2% DDM, and 10 mM imidazole). The column was successively washed with 2 CV of EB, 2 CV of EB with 0.5 M NaCl, 2 CV of EB with 10 mM imidazole, 2 CV of EB with 30 mM imidazole, and 2 CV of EB with 40 mM imidazole. The protein was eluted with EB containing 300 mM imidazole.

The imidazole was removed using a desalting PD10 column, and the buffer of the eluted protein was exchanged with 50 mM Tris-HCl, pH 8, 100 mM NaCl, 10% glycerol, and 0.2% DDM.

#### Reconstitution of BmrA

The protein was diluted to 0.2 mg·mL^–1^ with 50 mM Tris-HCl, pH 8.0, 100 mM NaCl, and 10% glycerol and mixed with a homemade preparation of *B. subtilis* lipids (with a lipid-to-protein ratio (M/M) of 0.5) solubilized in Triton X-100 with a molar ratio of 10:1 and incubated for 1 h. The DDM and Triton X-100 were eliminated by dialysis with Bio-beads (BioRad) in the dialysis solution during 9 days,^31^ and each dialysis bag containing 40 mL of protein solution was incubated in 5 L beakers containing buffer (50 mM Tris-HCl, pH 8.0, 100 mM NaCl, 10% glycerol).

For the preparation of the BmrA:ADP:Mg:Vi complexes, the protein (0.2 mg·mL^–1^), after 9 days of dialysis, was incubated with 1 mM Na_3_VO_4_ during 5 min, then 10 mM ATP and 10 mM Mg^2+^ (corresponding to a 3300:1 nucleotide/protein ratio (mol/mol) and displaying >90% saturation based on a measured *K*_d_ value in proteoliposomes^28^) were added and the incubation was pursued for 1 h at room temperature. The homogeneity of the vanadate sample was previously verified by a proteolysis resistance test.^32^ The gel quantification was performed using software ImageJ.^33^

For the BmrA-E504A:ATP:Mg and BmrA:ADP:Mg complexes, the proteins (0.2 mg·mL^–1^) were incubated with 10 mM ATP and 10 mM Mg^2+^ during 1 h at room temperature. All nucleotides were used in the presence of Mg^2+^ in a 1:1 (mol/mol) ratio.

The protein in lipids was sedimented into the MAS–NMR rotor by 120 000*g* centrifugation (30 min at 4 °C) using home-build tools.

#### NanoDSF Experiments

Proteoliposomes of BmrA WT or E504A were analyzed by nano-differential-scanning fluorimetry (nanoDSF). Thermal denaturation assays were performed using the Prometheus NT.48 instrument and analyzed using PR.thermocontrol V2.0.4. software (Nanotemper technologies, DE). BmrA WT or E504A reconstituted into proteoliposomes were used at 0.2 mg/mL, as described^32,34^ and supplemented with 10 mM ATP and/or 10 mM ADP, 10 mM MgCl_2_, and 1 mM Vi, when specified. Samples were incubated for 15 min at room temperature after the addition of ligands before analysis. The capillaries were then filled with 10 μL of the sample mixture and placed on the sample holder. A temperature gradient of 1 °C/min from 25 to 95 °C was applied, and the intrinsic protein fluorescence at 330 and 350 nm was recorded. The ratio of fluorescence intensity at 350/330 nm was used to determine the melting temperatures.

#### Solid-State NMR Experiments

^13^C solid-state NMR spectra were acquired at 20.0 T static magnetic field strength using a 3.2 mm Bruker Biospin “E-free” triple-resonance probe.^35^^31^P solid-state NMR spectra were acquired at 11.7 T static magnetic field strength using a Bruker 3.2 mm triple-resonance MAS probe. All ^13^C and ^31^P experiments were recorded at a spinning frequency of 17.0 kHz. The two-dimensional (2D) spectra were processed with software TOPSPIN (version 3.5, Bruker Biospin) with a shifted (SSB = 2.0 and 3.0 for ^31^P and ^13^C 2D spectra, respectively) sine-squared apodization function. Automated baseline correction to order five in the indirect and direct dimensions was applied. The sample temperature was set to 278 K as determined by the water proton chemical-shift value.^36^ All spectra were analyzed with software CcpNmr^37^ and referenced to 4,4-dimethyl-4-silapentane-1-sulfonic acid (DSS).^36^^31^P CPMAS and ^31^P–^31^P DARR spectra^38^ were collected for all complexes. ^13^C–^13^C DARR spectra shown in the Discussion section are from our previous work.^27^

## Results

### ADP Binds Weakly to BmrA as Revealed by the Observation of Chemical Exchange in the NMR Spectra

**Figure 2:**
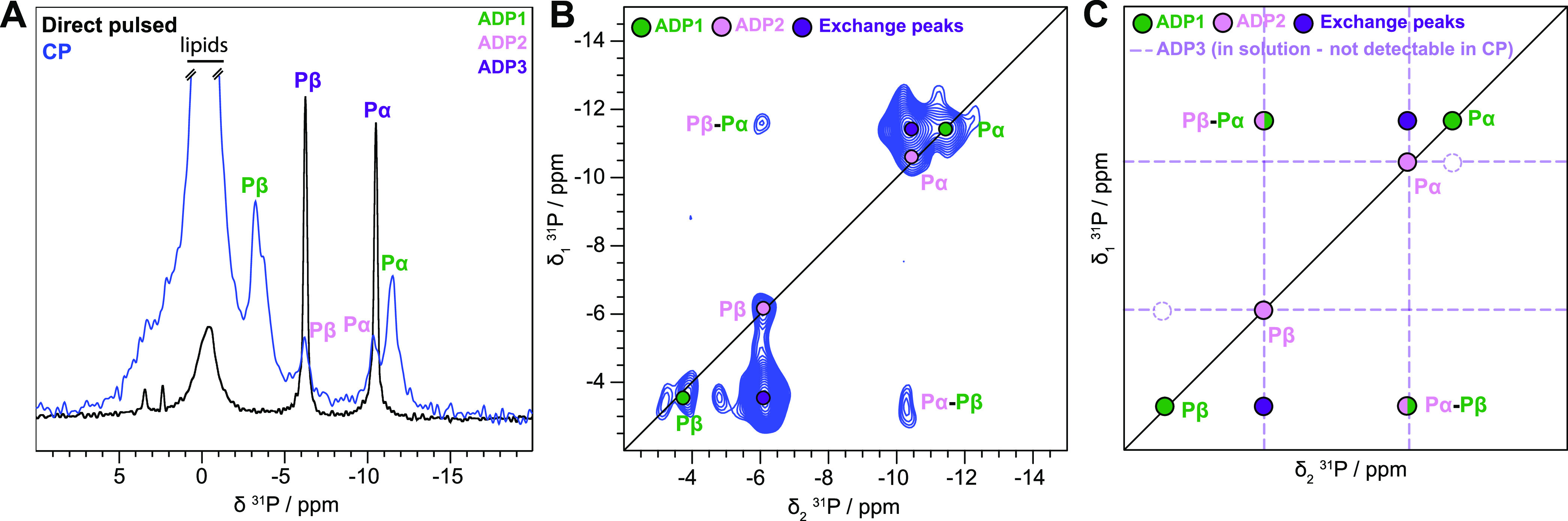
Different ADP species detected in BmrA:ADP. (A) ^31^P cross-polarization spectrum (blue) and direct-pulsed spectrum (black) of BmrA:ADP, showing bound and free ADP species, respectively. The one-dimensional (1D) CP spectrum of BmrA:ADP was taken from Lacabanne et al.^32^-Copyright 2020 Lacabanne http://creativecommons.org/licenses/by/4.0/. (B) ^31^P–^31^P 150 ms DARR correlation spectrum of BmrA:ADP. (C) Schematic representation of the experimental spectrum shown in panel (B).

We have performed ^31^P solid-state NMR experiments to probe the binding of ADP to BmrA. The ^31^P nucleotide signals benefit from the high sensitivity of the ^31^P chemical-shift values to small changes in the chemical environment and conformation.^32^Figure 2A shows the corresponding spectra of BmrA incubated with ADP (ADP/Mg^2+^). While the cross-polarization (CP) spectrum shows two different kinds of immobilized and therefore bound nucleotides (referred to as ADP1, green, and ADP2, pink), the direct-pulsed spectrum recorded with a short repetition time shows the unbound nucleotides present in the supernatant of the NMR rotor^39^ (referred to as ADP3, purple). ADP2 and ADP3 show very similar chemical shifts, which indicates that the ADP2 molecule is most likely barely impacted by binding to the protein, and thus presumably represents a species loosely bound to the NBD. The shifts of bound ADP1 differ significantly from those of ADP2 and ADP3, and the ADP1 resonances are much more intense than the ones of ADP2, which are also visible in the CP spectra, supporting their assignments to the same species.

To establish connectivities between ^31^P spins within the bound ADP molecules, we recorded a ^31^P–^31^P 2D exchange spectrum. In general, the polarization-transfer process in such a spectrum can be either due to chemical exchange, spin diffusion, or cross-relaxation (nuclear Overhauser effect, NOE). During the 150 ms mixing time, continuous-wave irradiation of the protons was applied to facilitate dipolar-assisted rotational resonance (DARR)^38^ to accelerate spin diffusion. The proton irradiation does not significantly influence the exchange and NOE mechanisms. The spectrum is shown in Figure 2B. The ADP1 Pβ resonance shows an intense cross-peak with the Pβ resonance of ADP2/3 (the same observation is made for the Pα resonance), Figure 2B,C. Interestingly, the spectrum is highly asymmetric, e.g., no back-transfer from ADP2/3 to ADP1 is observed, and the off-diagonal peak is five times more intense than the diagonal peaks. This clearly points to an exchange process with a nonequilibrium initial magnetization at the beginning of the mixing period. The initial CP step only selects immobilized nucleotides (ADP1/2), while ADP3 in solution cannot be cross-polarized and has no initial polarization at the start of the mixing time. The intense asymmetric Pβ–Pβ and Pα–Pα correlation peaks (purple circles in Figure 2C) indicate chemical exchange with a species, which is not excited by the initial CP step, i.e., the free ADP3. We note that there is no intramolecular polarization transfer between ADP1 Pα and Pβ, indicating that the exchange is faster than the intramolecular spin diffusion processes (Figure 2B,C). A second, much weaker pair of crosscorrelation peaks is, however, observed between ADP1 Pβ–ADP2/3 Pα and ADP1 Pα–ADP2/3 Pβ, Figure 2B,C. This step can obviously not be chemical exchange since it connects Pα and Pβ of two different ADP molecules. Again, for the back-transfer, no off-diagonal peak is observed. The cross-peak is thus caused by a two-step process of chemical exchange and Pα–Pβ ADP2 spin diffusion or by transferred Pα–Pβ NOE^40^ between the Pα–Pβ of ADP3. The latter would require ADP3 to be transiently bound to the protein, a state that indeed may be identical to ADP2. Altogether, the ^31^P spectrum highlights the transient binding of ADP to BmrA, which is exchanging with unbound ADP.

### ADP:Vi Is Tightly Bound to BmrA in One of the Two Binding Sites

**Figure 3:**
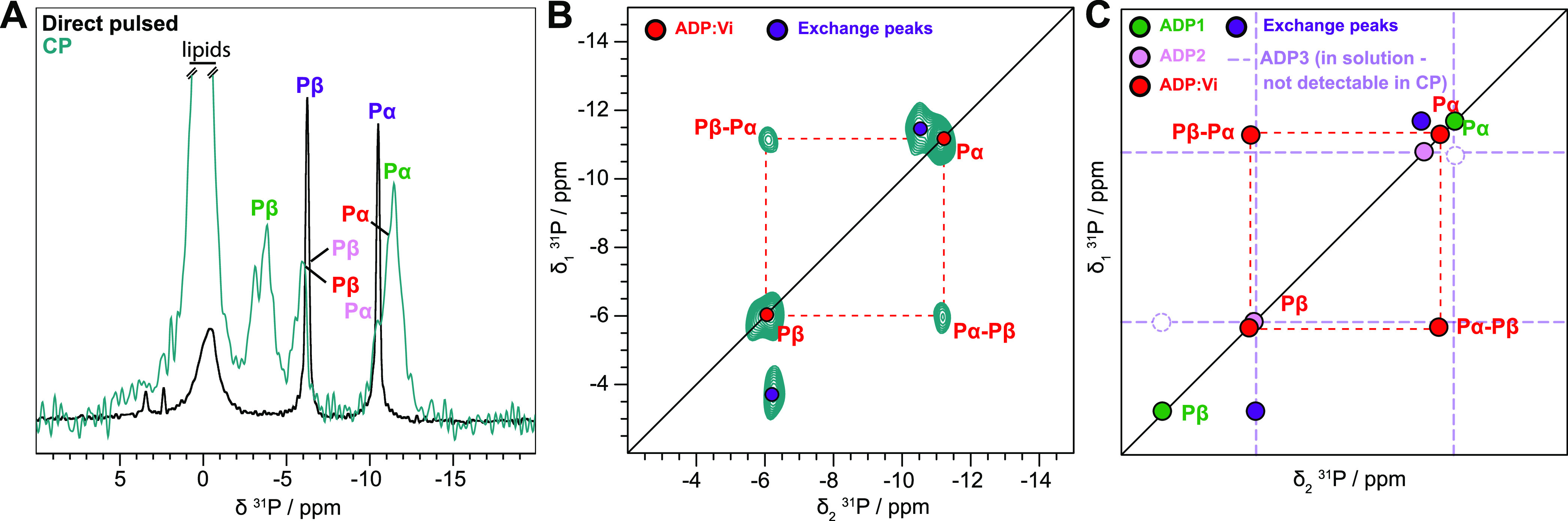
ADP:Vi is tightly bound to BmrA. (A) ^31^P cross-polarization spectrum (green) and direct-pulsed spectrum (black) of BmrA:ADP:Vi. The 1D CP spectrum of BmrA:ADP:Vi was taken from Lacabanne et al.^32^-Copyright 2020 Lacabanne http://creativecommons.org/licenses/by/4.0/. (B) ^31^P–^31^P 150 ms DARR correlation spectrum of BmrA:ADP:Vi. (C) Schematic representation of the experimental spectrum shown in panel (B).

We next incubated BmrA with ATP (ATP/Mg^2+^) and vanadate (Vi) to trap the transporter in the transition state of ATP hydrolysis.^32^ We have previously described that during this incubation step, ATP hydrolysis occurs and the protein switches its conformation from the inward- to the outward-facing state.^27^Figure 3A displays the CP- and direct-pulsed ^31^P spectra upon incubation of BmrA with ATP:Vi. An additional resonance is observed in the CP spectrum compared to the previously described spectrum of BmrA:ADP. We attribute this signal to ADP:Vi; indeed, ^31^P chemical-shift changes observed upon Vi binding are reported to be quite small.^41^ This assignment is confirmed by the ^31^P–^31^P DARR/exchange spectrum in Figure 3B, where a symmetric cross-peak is observed for this species, consistent with DARR polarization transfer between the ^31^P spins within the same ADP:Vi molecule, as expected for tightly bound ADP:Vi. This is further corroborated by gently washing the sample with a buffer solution since the remaining resonances visible in the ^31^P spectrum are the ones assigned to strongly bound ADP:Vi (Figures S1A and 1B). Interestingly, the spectrum still displays the asymmetric peak pattern attributed to chemical exchange between ADP1 and ADP2/3, similar to that of BmrA:ADP, except that some peaks remain invisible (e.g., the Pα–Pβ correlations), most likely due to a lower signal-to-noise ratio. Altogether, the ^31^P spectra support the assumption that the binding of ADP:Vi takes place in one NBD, while the second NBD is occupied by weakly bound ADP showing the same chemical-exchange features as described above for BmrA:ADP (Figure 2) and as summarized in Figure 3C.

### Prehydrolytic State Trapped in the Catalytic Mutant E504A

**Figure 4:**
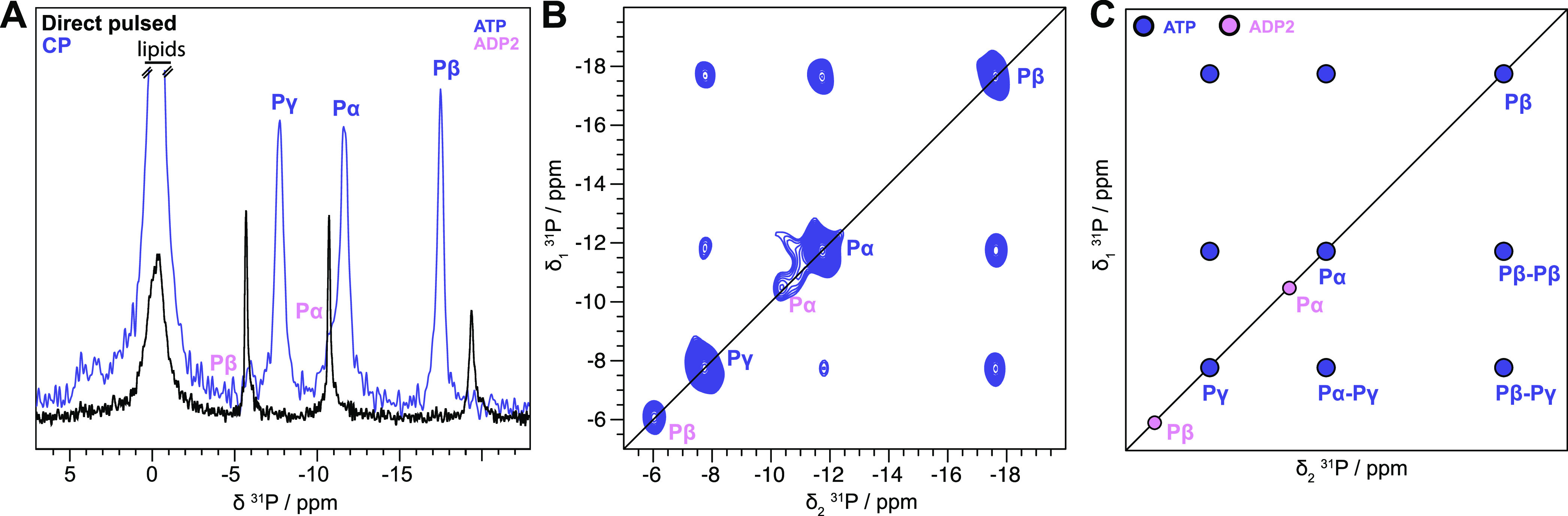
Hydrolytic-deficient E504A mutant binds ATP in the two NBDs. (A) ^31^P cross-polarization spectrum (pale purple) and direct-pulsed spectrum (black) of BmrA-E504A:ATP. The 1D CP spectrum of BmrA-E504A:ATP was taken from Lacabanne et al.^32^—Copyright 2020 Lacabanne http://creativecommons.org/licenses/by/4.0/. (B) ^31^P–^31^P 150 ms DARR correlation spectrum of BmrA-E504A:ATP revealing no chemical exchange of bound ATP (pale purple). A small fraction of hydrolyzed ADP can be observed (ADP2, pink). (C) Schematic representation of the experimental spectrum shown in panel (B).

ATP hydrolysis is strongly impaired in the BmrA mutant E504A, in which the catalytic glutamate adjacent to the Walker B motif is mutated.^32,34,42^ Consistent with its biochemical characterization and its recently solved ATP-bound 3D structure,^34,42,43^ the ^31^P CP spectrum indicates that ATP is bound to the mutant (Figure 4A). In contrast to the ADP:Vi-bound state described above, no asymmetry is observed in the 2D ^31^P–^31^P 150 ms DARR/exchange correlation spectrum (Figure 4B) in which the expected connectivity pattern for the triphosphate based on spin diffusion is observed. A ^31^P–^31^P 150 ms DARR spectrum was recorded during 2.5 days, and some weak signal of loosely bound ADP can be observed (pink) in the spectrum, suggesting that very slow hydrolysis could still occur in this mutant (Figure 4B). The analysis of the spectrum is summarized in Figure 4C.

### Prehydrolytic State is More Stable than the Transition State

**Figure 5:**
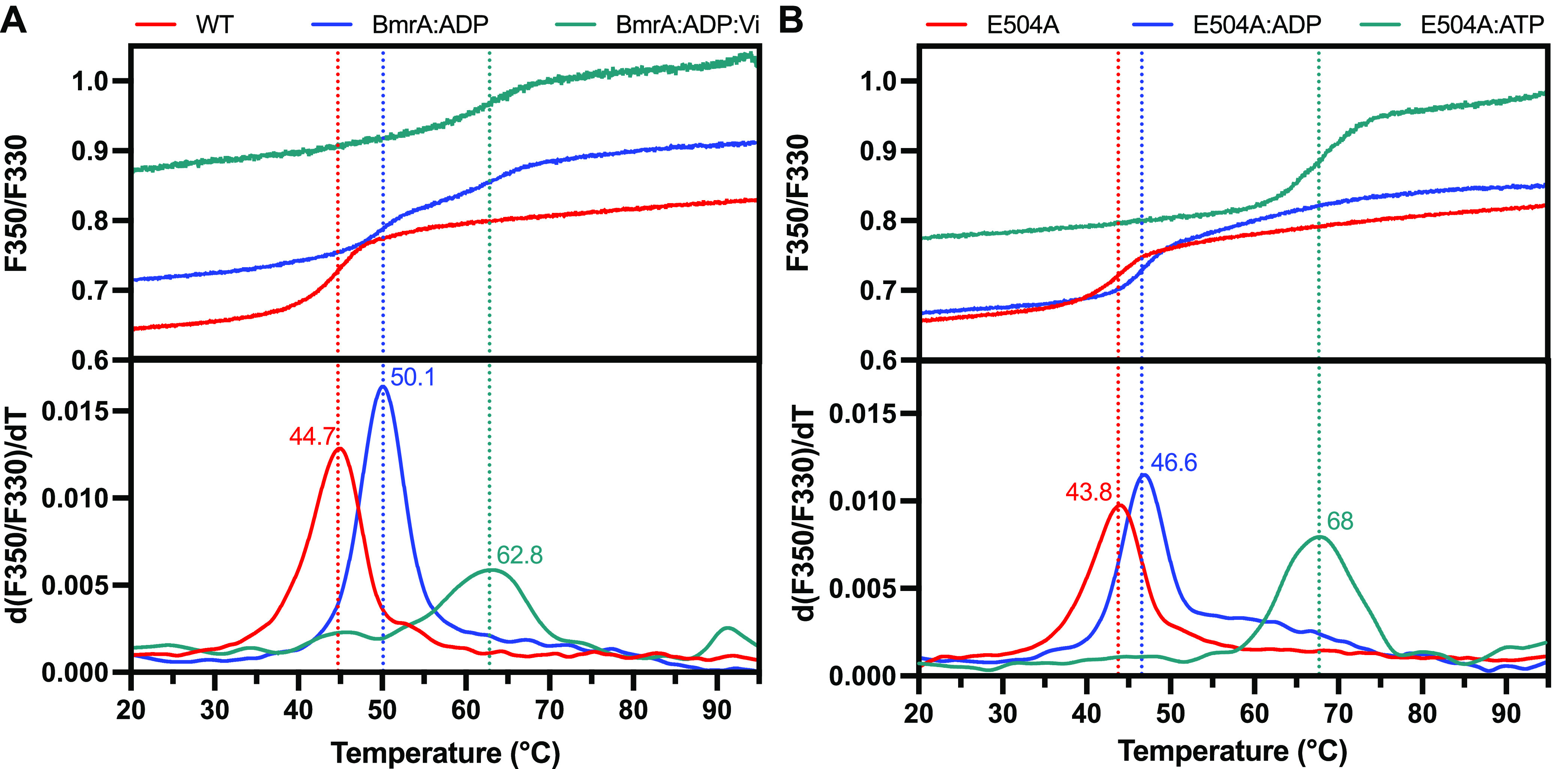
Thermostability of BmrA probed by nanoDSF measurements. Unfolding curves (top panel) and derivatives of the unfolding curves with the apparent melting temperatures (bottom panel) of (A) BmrA and (B) BmrA-E504A in the apo state (red lines) or in the presence of ADP (blue lines), ADP:Vi (green line, panel A), or ATP (green line, panel B).

The thermostability of BmrA in the presence of nucleotides was probed by nano-differential-scanning fluorimetry (nanoDSF). BmrA and BmrA-E504A display typical protein melting curves of folded proteins with very similar apparent melting temperatures of ∼45 °C (Figure 5A) and 44 °C (Figure 5B), respectively. In the presence of ADP (ADP/Mg^2+^), significant shifts of +5 °C (WT, Figure 5A) and +3 °C (BmrA-E504A, Figure 5B) in the apparent melting temperatures were observed. However, the presence of ATP (ATP/Mg^2+^) and vanadate leads to an enhanced temperature stability with a large shift of the melting temperature of WT BmrA by +18 °C (Figure 5A). Surprisingly, in the presence of ATP, the melting temperature of BmrA-E504A increases by 26 °C (Figure 5B) and is thus 8 °C higher than with vanadate, reflecting apparently an even higher stability. In contrast to some other ABC transporters, addition of ADP:Vi instead of ATP:Vi does not induce the conformational changes uniformly (Figure S7), and therefore the addition of ATP in the presence of Mg^2+^ is required to reach the fully trapped ADP:Vi-bound state with a high degree of homogeneity.^20,44^

## Discussion

**Figure 6:**
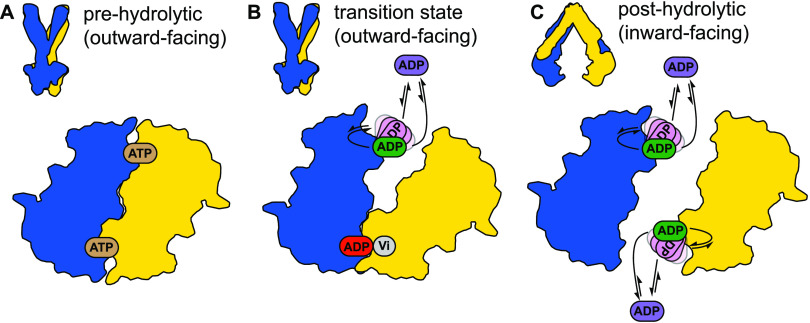
Nucleotide-binding models as deduced from the NMR data and assuming that ADP is in three different states. (A) Symmetric ATP binding as revealed by the E504A mutant in the presence of ATP, (B) asymmetric ADP binding as observed for the ADP:Vi-bound state (transition state) of the wild-type transporter, and (C) symmetric ADP binding observed for BmrA:ADP (posthydrolytic state).

Our spectroscopic observations yielded new mechanistic insights into ATP hydrolysis by BmrA. First, in the presence of ATP, the spectrum shows a homogeneous ATP-bound protein population. This observation leads to the conclusion that ATP is tightly bound to both NBDs (Figure 6A). This finding is supported by the recently published X-ray and cryo-EM structures of BmrA-E504A:ATP/Mg, where the two ATP-binding sites of the mutant are occupied by ATP:Mg^2+^.^42^ In the case of BmrA:ADP, chemical exchange is observed in the ^31^P exchange spectra. The nonequilibrium magnetization at the beginning of the mixing time is responsible for the asymmetric ^31^P–^31^P 2D spectra. ADP in three different chemical states is involved in the exchange, namely, (i) bound ADP that is sufficiently immobilized in the ATP-binding site so that CP is efficient and can be detected in ^31^P CP experiments; (ii) loosely bound ADP (e.g., possibly ADP is retained only by the Walker A motif but other motifs are not fully engaged in the stabilization of the nucleotide) that gives only a weak ^31^P CP signal; and (iii) free ADP in solution, which is not detected in ^31^P CP (Figure 6C). These states could be connected either by two subsequent two-site exchange processes or by a three-site exchange. Numerical simulations of a three-site chemical-exchange map (Figure S2) based on the McConnell equations^45^ are reported in the Supporting Materials section. Asymmetric three-site exchange spectra were for instance also recently reported in *T*_2_–*T*_2_ exchange experiments.^46^ Based on a recent study using the small-angle neutron scattering (SANS) technique, the BmrA ADP-bound state of BmrA seems to adopt exclusively an inward-facing conformation with the two NBDs fully separated.^43^

While in the BmrA:ADP:Vi-bound state we still observe the exchange phenomenon as in the ADP-bound state, it differs from the latter since the ADP:Vi is tightly trapped in the homodimer, indicating a mixed conformation with one NBD tightly binding the ADP:Vi and one NBD sufficiently open to allow nucleotide exchange (Figure 6B). This observation was confirmed by the removal of the ADP in excess with a gentle washing step, after which only ADP:Vi tightly bound to the protein remains detectable on the ^31^P 1D spectra (Figure S1).

Moreover, the thermostability of BmrA probed by nanoDSF highlights differences between the outward-facing state obtained using vanadate (with BmrA WT) and using ATP (with the catalytic-inactive mutant E504A). The small shift in the melting temperature by a few degrees (5 °C, BmrA, Figures 5A, and 3 °C, BmrA-E504A, Figure 5B) confirmed that ADP binds to the ATP-binding site of both proteins, but that it does not induce a drastic conformational change. Indeed, this range of temperature shift is typical for a substrate- or analogue-binding event, which mildly increases the melting temperature,^47^ and a similar result was recently reported for BmrA in detergent.^43^ In the presence of vanadate, the conformation of the protein changes to the outward-facing state leading to a more thermostable conformation, strongly increasing the melting temperature by 18 °C. This temperature shift is characteristic of large conformational changes or binding of a very strong inhibitor.^47,48^ Interestingly, with BmrA-E504A, the presence of ATP increases the apparent melting temperature by 26 °C. On the other hand, since a single ATP site is occupied by ADP:Mg:Vi while the second site has only a bound ADP, this heterogeneity might be reflected by the lower *T*_m_ as compared to the ATP-bound state of the E504A mutant.

**Figure 7:**
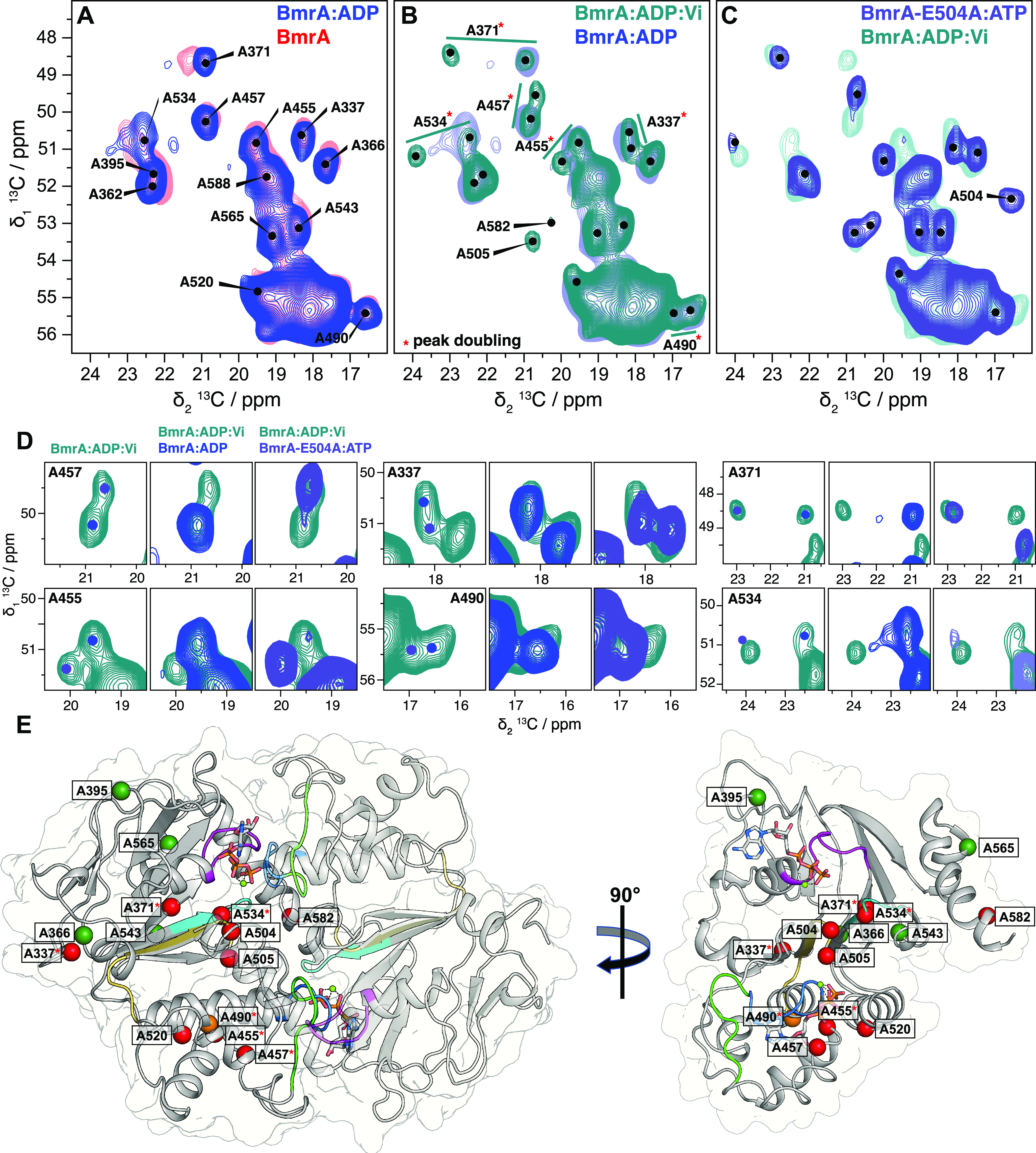
BmrA conformation probed by ^13^C-detected 2D DARR experiments. Alanine region spectral fingerprint of (A) ADP-bound state of the WT protein overlaid with the apo state, (B) transition state of the WT:ADP:Vi overlaid with the ADP-bound state, and (C) prehydrolytic state using the E504A mutant overlaid with the WT:ADP:Vi. (D) Zooms of peak splitting from WT:ADP:Vi compared with WT:ADP and E504A:ATP. Examples of peak splitting from other regions of the spectrum are presented in Figure S4. BmrA, BmrA:ADP:Vi, and BmrA-E504A:ATP spectra were adapted from data previously recorded (Lacabanne et al.^27^—Copyright 2019 Lacabanne http://creativecommons.org/licenses/by/4.0/). The colored dots indicate peak maxima in the BmrA:ADP (light blue) and BmrA-E504A:ATP spectra (gray). Resonance assignments were transferred from solution-state NMR assignments obtained on the isolated NBD.^49^ (E) View of the NBDs of BmrA-E504A:ATP in the full-length structure (pdb 7OW8); the different motifs are highlighted in different colors: the X-loop (470-TEVGERG-476) in green, the Walker A motif (374-GPSGGKT-381) in magenta, the Walker B motif (496-ILMLDE-504) in dark yellow, the ABC signature (477-LSGGQ-483) in blue, and the H-loop (532-AHR-536) in cyan. Alanine residues resolved in the 2D NMR spectra are shown as colored spheres according to the chemical-shift perturbations determined between BmrA-apo and BmrA-E504A:ATP: red CSP > 0.6 ppm (or appearing peaks: A504, A505, A582), orange CSP > 0.2 ppm, and dark green CSP < 0.2 ppm. Resonances displaying peak doubling are indicated by a red star.

While ^31^P spectra confirmed by nanoDSF experiments revealed the nature of the bound nucleotide, ^13^C spectroscopy allows analyzing the conformation of the protein itself. In light of the identification of two different ATP-binding modes in the transition-state mimic, described in the Results section, revealed by ^31^P-detected NMR spectroscopy and nanoDSF, we revisited the ^13^C-detected spectra described previously.^27^ This new analysis was also enabled by the recent publication of the near-complete backbone assignment of the NBD of BmrA in the presence of ADP as obtained by solution-state NMR.^49^ Indeed, Hellmich and co-workers have shown that the isolated BmrA NBD can be studied in solution since it is stable and conserves its structural integrity and ability to interact with nucleotides.^49^ Moreover, in the case of BmrA and LmrA, the NBDs are monomeric in the absence of the TMDs, making them particularly suitable for solution NMR studies.^24,49^ It has been shown in several cases that solution-state NMR chemical shifts can be transferred reasonably well to the solid-state NMR, assuming that the structural features are maintained.^32,50^ To resolve the remaining ambiguities and to crosscheck the transferred assignments, 2D DARR experiments with a long mixing time (200 ms) were employed in our case (Figure S3). These experiments allow probing long-range correlations. The available solution-state NMR shifts allowed us to transfer many assignments and thereby also correct previous tentative resonance assignments.^27^Figure 7A–C shows the alanine region of 2D DARR spectra of the prehydrolytic, transition, and posthydrolytic states (mimicked by BmrA-E504A:ATP, BmrA:ADP:Vi, and BmrA:ADP, respectively) previously recorded.^27^ From this region, two important observations can be made: first, chemical shifts change quite substantially between the prehydrolytic state (BmrA-E504A:ATP) and the posthydrolytic state (BmrA:ADP), and second, as stated previously,^27^ many additional peaks are observed in the BmrA:ADP:Vi spectrum. These resonances are located in the NBDs as shown in previously reported paramagnetic NMR experiments.^27^Figure 7 shows overlays of the four spectra.

It can be seen that in the ADP-bound state, the resonances of the NBD residues are not drastically different from the apo state (Figure 7A); the rather small ^13^C chemical-shift perturbations (CSPs) of less than 0.3 ppm are indicative of ADP binding not causing drastic conformational changes. This illustrates that the apo protein and the ADP-bound state are still in the inward-facing conformation as recently suggested from SANS experiments.^43^ The small CSPs observed (Figures 7A, S4, and S5A,B for the entire aliphatic region) reflect complete saturation of the NBDs with ADP and indicate minor conformational changes upon ADP binding. A pronounced shift is, for instance, observed for A371 close to the Walker A motif (Figure 7A), supporting the assumption that the ADP still binds to the expected ATP-binding site. This is consistent with the rather small *T*_m_ increase observed by nanoDSF in the presence of ADP alone. In contrast, for BmrA-E504A:ATP, the spectrum shows not only small CSPs <0.2 ppm but also quite large CSPs (0.6 up to 3 ppm) (Figure 7C–E).

Finally, in agreement with the results obtained from the ^31^P spectra, it becomes now clear that the BmrA:ADP:Vi spectrum is actually close to being the sum of the BmrA-E504A:ATP and BmrA:ADP spectra, see the resonance intensities (Figure S6), i.e., that part of its resonances reflects each state, resulting in peak doubling. BmrA:ADP:Vi thus clearly represents an asymmetric state. One NBD is occupied with a tightly bound ADP:Vi (no chemical exchange is observed in ^31^P spectra), as observed in BmrA-E504A:ATP, and the other NBD weakly binds ADP, as observed in BmrA:ADP. The second NBD is affected by the same exchange process described above for the ADP-bound state. The peaks with small CSPs resulting from ADP binding do not display peak doubling (see Figure S4 for other examples); however, peak doubling with large CSPs is caused by one NBD occupied with ADP:Vi and one NBD with ADP only, as highlighted in Figure 7D (see Figure S4 for other examples). This is in good agreement with the conclusions drawn from the ^31^P data but contradicts in part our previous interpretation, namely, that rigidification of the NBD was responsible for the subset of peaks appearing in BmrA:ADP:Vi.^27^ Based on the current ^31^P results, we can now assign this to peak doubling, caused by the two differently occupied NBDs in BmrA:ADP:Vi. Figure 7E shows the alanine residues assigned in Figure 7A,B plotted on the cryo-EM structure of the E504A mutant in complex with ATP/Mg^2+^. All of them are located in the NBDs.

The nanoDSF shown in Figure S7 allows us to rule out the presence of two different protein–nucleotide complex populations (i.e., one bound exclusively with ADP:vanadate and the other one bound exclusively with ADP) upon incubation with ATP and Vi. Indeed, when we instead started the incubation directly with ADP:Vi, we observed two *T*_m_ that most likely reflect two different BmrA populations, one with two bound ADP/Mg (*T*_m_ = 55.1 °C) and one with bound ADP:Vi (one or two, *T*_m_ = 62.8 °C). A proteolysis resistance assay was also performed on the BmrA:ADP:Vi sample (trapped state induced with ATP incubation) to confirm this result, as described in a previous study^27^ and Figure S8. In contrast to the inward-facing conformation, BmrA in the outward-facing conformation is highly resistant toward limited proteolysis by trypsin. The Vi-trapped state is resistant toward proteolysis digestion showing that the whole sample is homogeneous and constituted by one population displaying an outward-facing conformation.^27^ We estimate that only 10% of the sample returned to the inward-facing form after 36 h, which is the duration of the ^13^C-detected 2D DARR (Figure S8).

Together, the NMR data thus clearly reveal an asymmetric structure of the two ATP-binding sites in the BmrA:ADP:Vi transition-state mimic, in which one site occupied by ADP:Vi is not accessible for an exchange process, whereas the second site is sufficiently open to allow the exchange with nucleotides from the solvent (as shown in Figure 6B). This tight trapping in the presence of vanadate of a single ADP molecule per BmrA dimer is in agreement with the trapping stoichiometry found previously with this transporter and using a radioactive ATP analogue.^42^ Likewise, the same stoichiometry of trapping was reported for other ABC transporters in the presence of vanadate, such as the P-glycoprotein,^18^ LmrA,^19^ or the maltose transporter.^20^ In contrast, the 3D structures of the maltose transporter and MsbA solved in the presence of ATP:Vi strongly support the presence of two ADP:Vi bound per transporter.^51^ It was argued that this difference in stoichiometry could be due to a nonequilibrium process in the biochemical experiments where the free nucleotides (or analogues) had to be removed from the sample. In the present work, however, the equilibrium between the bound and free ADP:Vi was maintained, in a situation similar to that found in cryo-EM or crystallography for the 3D structures mentioned above, but the results clearly show the tight binding of a single ADP:Vi complex to a BmrA dimer.

In the ATP-bound state (trapped using the mutant E504A), we have neither detected any exchange in the ^31^P spectra nor any peak splitting in the ^13^C NMR spectra,^32^ and thus we can conclude that a symmetric coordination of ATP by the two ATP-binding sites takes place. This is supported by the ^13^C peak intensities of the newly appearing peaks of the NBD, which are roughly twice as intense as those for BmrA:ADP:Vi in which only one site is saturated by ADP:Vi (Figure S6).

**Figure 8:**
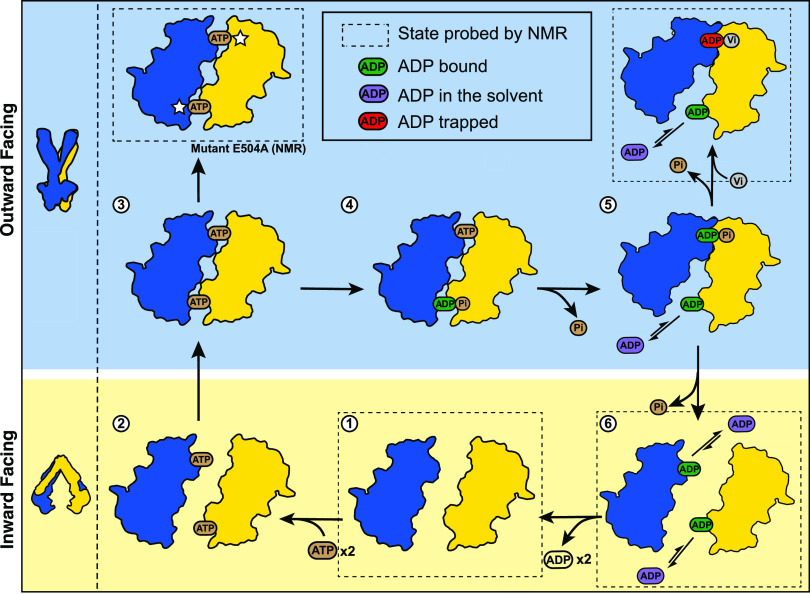
Scheme of ATP hydrolysis by the multidrug ABC transporter BmrA. The two BmrA monomers are shown in yellow and blue and the NBDs are shown in contours. Dashed squares indicated the states that were studied by NMR. The E504A mutation is indicated by a white star. For a description of the cycle, please see the text. One can see that the process adopted by BmrA results in a mixture of the two alternative models shown in Figure 1.

The combination of ^13^C and ^31^P NMR thus yields further insight into the detailed mechanism of ATP hydrolysis in the ABC transporter BmrA, leading to the proposed scheme shown in Figure 8. Initially, the protein is in a symmetric apo state (step **1**), which is an inward-facing, open conformation. ATP binding (step **2**) induces a tight dimerization of the two NBDs and switches the transporter to the outward-facing conformation (step **3**). This state was experimentally characterized by observing the E504A mutant that is trapped in this conformation and is unable to proceed (step **4**). The symmetric binding of ATP to both NBDs and the switch to the outward-facing conformation have also been observed in the X-ray and cryo-EM structure of the E504A mutant in complex with ATP/Mg^2+^.^42^ The ^13^C NMR spectra between (**1**) and (**3**) show significant chemical-shift differences. For the WT protein, ATP hydrolysis from (**3**) to (**4**) proceeds. From state **4**, (i) the two NBDs could dissociate (if ATP hydrolysis in the second ATP site is slower than the dissociation); here the hydrolysis of one ATP molecule could be sufficient to destabilize the dimer; or (ii) directly proceed to state **5** (if ATP hydrolysis on the second state is faster than the dissociation); here two ATP molecules are hydrolyzed to disrupt the dimer. The addition of vanadate allows trapping of the transition state for ATP hydrolysis (state 5 in complex with ADP:Vi). The ADP coordinated to Vi is shown by NMR to be tightly bound (trapped) and not exchanging with free ADP, while the ADP on the more “accessible” side of the dimer is bound and exchanges with free ADP on the millisecond time scale. The comparison of the ^13^C NMR spectra of state (**3**) with (**5**) indicates that one site (with bound ADP) adopts a similar conformation to **(1)**, the apoprotein, while the second ATP site (with tightly bound ADP:Vi) has a conformation that resembles (**3**). The final step in this cycle leads again to a symmetric state, to the inward-facing posthydrolytic state (**6**) with two ADPs bound. ^31^P NMR reveals an exchange process between bound ADP and ADP in solution for both NBD domains on a millisecond time scale. The ^13^C spectra of the ADP and the apo protein are quite similar, leading us, in combination with limited proteolysis data, to the conclusion that the protein adopts already the inward-facing conformation, in agreement with SANS data obtained for BmrA in detergent.^43^

## Conclusions

In summary, our NMR study did not only allow us to characterize conformational changes during ATP hydrolysis but also unraveled dynamical exchange phenomena fundamental for the functioning of such protein engines. The asymmetry in the NBDs occurring during ATP hydrolysis seems to be a key feature of ATP hydrolysis in BmrA and is only observed in the transition state, whereas the pre- and posthydrolytic states are quite symmetric as shown by solid-state NMR herein and for the prehydrolytic state additionally by cryo-EM and X-ray crystallography.^42^ Our studies also pinpoint the importance of studying the whole ATP-hydrolysis cycle, which is particularly feasible by solid-state NMR due to the simple sedimentation process used for NMR sample preparation.

## Supporting Information Available

Supporting figures of additional NMR, nanoDSF, and proteolysis experiments; and numerical simulations of a three-site chemical-exchange map (PDF)

## References

[ref1] aDean M.; Rzhetsky A.; Allikmets R. The human ATP-binding cassette (ABC) transporter superfamily. Genome Res. 2001, 11, 1156–1166. 10.1101/gr.184901. 11435397

[ref2] aKerr I. D. Structure and association of ATP-binding cassette transporter nucleotide-binding domains. Biochim. Biophys. Acta, Biomembr. 2002, 1561, 47–64. 10.1016/S0304-4157(01)00008-9. 11988180

[ref3] Thomas C.; Tampé R. Structural and Mechanistic Principles of ABC Transporters. Annu. Rev. Biochem. 2020, 89, 605–636. 10.1146/annurev-biochem-011520-105201. 32569521

[ref4] Choi Y. H.; Yu A. M. ABC transporters in multidrug resistance and pharmacokinetics, and strategies for drug development. Curr. Pharm. Des. 2014, 20, 793–807. 10.2174/138161282005140214165212. 23688078 PMC6341993

[ref5] Robey R. W.; Pluchino K. M.; Hall M. D.; Fojo A. T.; Bates S. E.; Gottesman M. M. Revisiting the role of ABC transporters in multidrug-resistant cancer. Nat. Rev. Cancer 2018, 18, 452–464. 10.1038/s41568-018-0005-8. 29643473 PMC6622180

[ref6] Orelle C.; Mathieu K.; Jault J.-M. Multidrug ABC transporters in bacteria. Res. Microbiol. 2019, 170, 381–391. 10.1016/j.resmic.2019.06.001. 31251973

[ref7] Sun Y. L.; Patel A.; Kumar P.; Chen Z. S. Role of ABC transporters in cancer chemotherapy. Chin J Cancer 2012, 31, 51–57. 10.5732/cjc.011.10466. 22257384 PMC3777472

[ref8] Smith P. C.; Karpowich N.; Millen L.; Moody J. E.; Rosen J.; Thomas P. J.; Hunt J. F. ATP binding to the motor domain from an ABC transporter drives formation of a nucleotide sandwich dimer. Mol. Cell 2002, 10, 139–149. 10.1016/s1097-2765(02)00576-2. 12150914 PMC3516284

[ref9] Szöllősi D.; Rose-Sperling D.; Hellmich U. A.; Stockner T. Comparison of mechanistic transport cycle models of ABC exporters. Biochim. Biophys. Acta, Biomembr. 2018, 1860, 818–832. 10.1016/j.bbamem.2017.10.028. 29097275 PMC7610611

[ref10] Higgins C. F.; Linton K. J. The ATP switch model for ABC transporters. Nat Struct Mol Biol 2004, 11, 918–926. 10.1038/nsmb836. 15452563

[ref11] Janas E.; Hofacker M.; Chen M.; Gompf S.; van der Does C.; Tampé R. The ATP hydrolysis cycle of the nucleotide-binding domain of the mitochondrial ATP-binding cassette transporter Mdl1p. J. Biol. Chem. 2003, 278, 26862–26869. 10.1074/jbc.M301227200. 12746444

[ref12] George A. M.; Jones P. M. Perspectives on the structure-function of ABC transporters: the Switch and Constant Contact models. Prog. Biophys. Mol. Biol. 2012, 109, 95–107. 10.1016/j.pbiomolbio.2012.06.003. 22765920

[ref13] aZoghbi M. E.; Altenberg G. A. ATP binding to two sites is necessary for dimerization of nucleotide-binding domains of ABC proteins. Biochem. Biophys. Res. Commun 2014, 443, 97–102. 10.1016/j.bbrc.2013.11.050. 24269240 PMC3901029

[ref14] Zoghbi M. E.; Altenberg G. A. Hydrolysis at one of the two nucleotide-binding sites drives the dissociation of ATP-binding cassette nucleotide-binding domain dimers. J. Biol. Chem. 2013, 288, 34259–34265. 10.1074/jbc.M113.500371. 24129575 PMC3837166

[ref15] Lewinson O.; Orelle C.; Seeger M. A. Structures of ABC transporters: handle with care. FEBS Lett. 2020, 594, 3799–3814. 10.1002/1873-3468.13966. 33098660 PMC7756565

[ref16] Zaitseva J.; Oswald C.; Jumpertz T.; Jenewein S.; Wiedenmann A.; Holland I. B.; Schmitt L. A structural analysis of asymmetry required for catalytic activity of an ABC-ATPase domain dimer. EMBO J. 2006, 25, 3432–3443. 10.1038/sj.emboj.7601208. 16858415 PMC1523178

[ref17] aMittal A.; Böhm S.; Grütter M. G.; Bordignon E.; Seeger M. A. Asymmetry in the homodimeric ABC transporter MsbA recognized by a DARPin. J. Biol. Chem. 2012, 287, 20395–20406. 10.1074/jbc.M112.359794. 22523072 PMC3370220

[ref18] Tombline G.; Senior A. E. The occluded nucleotide conformation of p-glycoprotein. J Bioenerg. Biomembr. 2005, 37, 497–500. 10.1007/s10863-005-9498-4. 16691489

[ref19] van Veen H. W.; Margolles A.; Muller M.; Higgins C. F.; Konings W. N. The homodimeric ATP-binding cassette transporter LmrA mediates multidrug transport by an alternating two-site (two-cylinder engine) mechanism. EMBO J. 2000, 19, 2503–2514. 10.1093/emboj/19.11.2503. 10835349 PMC212756

[ref20] Sharma S.; Davidson A. L. Vanadate-induced trapping of nucleotides by purified maltose transport complex requires ATP hydrolysis. J. Bacteriol. 2000, 182, 6570–6576. 10.1128/JB.182.23.6570-6576.2000. 11073897 PMC111395

[ref21] aVerhalen B.; Dastvan R.; Thangapandian S.; Peskova Y.; Koteiche H. A.; Nakamoto R. K.; Tajkhorshid E.; McHaourab H. S. Energy transduction and alternating access of the mammalian ABC transporter P-glycoprotein. Nature 2017, 543, 738–741. 10.1038/nature21414. 28289287 PMC5558441

[ref22] Parcej D.; Tampé R. ABC proteins in antigen translocation and viral inhibition. Nat Chem Biol 2010, 6, 572–580. 10.1038/nchembio.410. 20644544

[ref23] Jones P. M.; George A. M. Mechanism of the ABC transporter ATPase domains: catalytic models and the biochemical and biophysical record. Crit. Rev. Biochem. Mol. Biol. 2013, 48, 39–50. 10.3109/10409238.2012.735644. 23131203

[ref24] Hellmich U. A.; Mönkemeyer L.; Velamakanni S.; van Veen H. W.; Glaubitz C. Effects of nucleotide binding to LmrA: A combined MAS-NMR and solution NMR study. Biochim. Biophys. Acta 2015, 1848, 3158–3165. 10.1016/j.bbamem.2015.10.003. 26449340

[ref25] aKaur H.; Lakatos A.; Spadaccini R.; Vogel R.; Hoffmann C.; Becker-Baldus J.; Ouari O.; Tordo P.; McHaourab H.; Glaubitz C. The ABC exporter MsbA probed by solid state NMR – challenges and opportunities. Biol. Chem. 2015, 396, 1135–1149. 10.1515/hsz-2015-0119. 25849794 PMC7906285

[ref26] Kunert B.; Gardiennet C.; Lacabanne D.; Calles-Garcia D.; Falson P.; Jault J.-M.; Meier B. H.; Penin F.; Böckmann A. Efficient and stable reconstitution of the ABC transporter BmrA for solid-state NMR studies. Front. Mol. Biol. 2014, 1, 5 10.3389/fmolb.2014.00005. PMC4428385 25988146

[ref27] Lacabanne D.; Orelle C.; Lecoq L.; Kunert B.; Chuilon C.; Wiegand T.; Ravaud S.; Jault J.-M.; Meier B. H.; Böckmann A. Flexible-to-rigid transition is central for substrate transport in the ABC transporter BmrA from Bacillus subtilis. Commun. Biol. 2019, 2, 149 10.1038/s42003-019-0390-x. 31044174 PMC6488656

[ref28] aSteinfels E.; Orelle C.; Fantino J. R.; Dalmas O.; Rigaud J. L.; Denizot F.; Di Pietro A.; Jault J. M. Characterization of YvcC (BmrA), a multidrug ABC transporter constitutively expressed in Bacillus subtilis. Biochemistry 2004, 43, 7491–7502. 10.1021/bi0362018. 15182191

[ref29] Lacabanne D.; Meier B. H.; Böckmann A. Selective labeling and unlabeling strategies in protein solid-state NMR spectroscopy. J. Biomol. NMR 2018, 71, 141–150. 10.1007/s10858-017-0156-z. 29197975

[ref30] Mathieu K.; Javed W.; Vallet S.; Lesterlin C.; Candusso M. P.; Ding F.; Xu X. N.; Ebel C.; Jault J. M.; Orelle C. Functionality of membrane proteins overexpressed and purified from E. coli is highly dependent upon the strain. Sci. Rep. 2019, 9, 2654 10.1038/s41598-019-39382-0. 30804404 PMC6390180

[ref31] Lacabanne D.; Lends A.; Danis C.; Kunert B.; Fogeron M.-L.; Jirasko V.; Chuilon C.; Lecoq L.; Orelle C.; Chaptal V.; et al. Gradient reconstitution of membrane proteins for solid-state NMR studies. J. Biomol. NMR 2017, 69, 81–91. 10.1007/s10858-017-0135-4. 28900789

[ref32] Lacabanne D.; Wiegand T.; Wili N.; Kozlova M. I.; Cadalbert R.; Klose D.; Mulkidjanian A. Y.; Meier B. H.; Böckmann A. ATP Analogues for Structural Investigations: Case Studies of a DnaB Helicase and an ABC Transporter. Molecules 2020, 25, 5268 10.3390/molecules25225268. PMC7698047 33198135

[ref33] Schneider C. A.; Rasband W. S.; Eliceiri K. W. NIH Image to ImageJ: 25 years of image analysis. Nat Methods 2012, 9, 671–675. 10.1038/nmeth.2089. 22930834 PMC5554542

[ref34] Orelle C.; Dalmas O.; Gros P.; Di Pietro A.; Jault J. M. The conserved glutamate residue adjacent to the Walker-B motif is the catalytic base for ATP hydrolysis in the ATP-binding cassette transporter BmrA. J. Biol. Chem. 2003, 278, 47002–47008. 10.1074/jbc.M308268200. 12968023

[ref35] Gor’kov P. L.; Witter R.; Chekmenev E. Y.; Nozirov F.; Fu R.; Brey W. W. Low-E probe for (19)F-(1)H NMR of dilute biological solids. J. Magn. Reson. 2007, 189, 182–189. 10.1016/j.jmr.2007.09.008. 17920316

[ref36] Lacabanne D.; Kunert B.; Gardiennet C.; Meier B. H.; Bo Ckmann A. Sample Preparation for Membrane Protein Structural Studies by Solid-State NMR. Methods Mol. Biol. 2017, 1635, 345–358. 10.1007/978-1-4939-7151-0_19. 28755379

[ref37] aFogh R.; Ionides J.; Ulrich E.; Boucher W.; Vranken W.; Linge J. P.; Habeck M.; Rieping W.; Bhat T. N.; Westbrook J.; et al. The CCPN project: an interim report on a data model for the NMR community. Nat Struct Biol, 2002, 9, 416–418. 10.1038/nsb0602-416. 12032555

[ref38] aTakegoshi K.; Nakamura S.; Terao T. 13C–1H dipolar-assisted rotational resonance in magic-angle spinning NMR. Chem. Phys. Lett. 2001, 344, 631–637. 10.1016/S0009-2614(01)00791-6.

[ref39] Wiegand T. A solid-state NMR tool box for the investigation of ATP-fueled protein engines. Prog. Nucl. Magn. Reson. Spectrosc. 2020, 117, 1–32. 10.1016/j.pnmrs.2020.02.001. 32471533

[ref40] Williamson M. P. The Transferred NOE, bookTitle= Modern Magnetic Resonance; Springer: Netherlands, 2006 10.1007/1-4020-3910-7_148.

[ref41] aRay B. D.; Moore J. M.; Rao B. D. 31P NMR studies of enzyme-bound substrate complexes of yeast 3-phosphoglycerate kinase: III. Two ADP binding sites and their Mg(II) affinity; effects of vanadate and arsenate on enzymic complexes with ADP and 3-P-glycerate. J. Inorg. Biochem. 1990, 40, 47–57. 10.1016/0162-0134(90)80039-z. 2283509

[ref42] Chaptal V.; Zampieri V.; Wiseman B.; Orelle C.; Martin J.; Nguyen K. A.; Gobet A.; Di Cesare M.; Magnard S.; Javed W.; et al. Substrate-bound and substrate-free outward-facing structures of a multidrug ABC exporter. Sci. Adv. 2022, 8, eabg9215 10.1126/sciadv.abg9215. 35080979 PMC8791611

[ref43] Javed W.; Vallet S.; Clement M. P.; Le Roy A.; Moulin M.; Härtlein M.; Breyton C.; Burlet-Schiltz O.; Marcoux J.; Orelle C.; et al. Structural insights into the catalytic cycle of a bacterial multidrug ABC efflux pump. J. Mol. Biol. 2022, 434, 167541 10.1016/j.jmb.2022.167541. 35292347

[ref44] Urbatsch I. L.; Tyndall G. A.; Tombline G.; Senior A. E. P-glycoprotein catalytic mechanism: studies of the ADP-vanadate inhibited state. J. Biol. Chem. 2003, 278, 23171–23179. 10.1074/jbc.M301957200. 12670938

[ref45] McConnell H. M. Reaction Rates by Nuclear Magnetic Resonance. J. Chem. Phys. 1958, 28, 430–431. 10.1063/1.1744152.

[ref46] Gao Y.; Blümich B. Analysis of three-site T2-T2 exchange NMR. J. Magn. Reson. 2020, 315, 106740 10.1016/j.jmr.2020.106740. 32438312

[ref47] Jaiquel Baron S.; King M. S.; Kunji E. R. S.; Schirris T. J. J. Characterization of drug-induced human mitochondrial ADP/ATP carrier inhibition. Theranostics 2021, 11, 5077–5091. 10.7150/thno.54936. 33859735 PMC8039944

[ref48] aHarborne S. P. D.; King M. S.; Kunji E. R. S. Thermostability Assays: a Generic and Versatile Tool for Studying the Functional and Structural Properties of Membrane Proteins in Detergents. Methods Mol. Biol. 2020, 2168, 105–121. 10.1007/978-1-0716-0724-4_5. 33582989

[ref49] Pérez Carrillo V. H.; Rose-Sperling D.; Tran M. A.; Wiedemann C.; Hellmich U. A. Backbone NMR assignment of the nucleotide binding domain of the Bacillus subtilis ABC multidrug transporter BmrA in the post-hydrolysis state. Biomol. NMR Assign. 2022, 16, 81–86. 10.1007/s12104-021-10063-2. 34988902 PMC9068644

[ref50] aBoudet J.; Devillier J. C.; Wiegand T.; Salmon L.; Meier B. H.; Lipps G.; Allain F. H. A Small Helical Bundle Prepares Primer Synthesis by Binding Two Nucleotides that Enhance Sequence-Specific Recognition of the DNA Template. Cell 2019, 176, 154–166.e113. 10.1016/j.cell.2018.11.031. 30595448

[ref51] aOldham M. L.; Chen J. Snapshots of the maltose transporter during ATP hydrolysis. Proc. Natl. Acad. Sci. U.S.A. 2011, 108, 15152–15156. 10.1073/pnas.1108858108. 21825153 PMC3174604

