# Supplementary material for: Solid-State NMR Reveals Asymmetric ATP Hydrolysis in the Multidrug ABC Transporter BmrA
Source: J Am Chem Soc. 2022 Jul 1;144(27):12431–42. doi: 10.1021/jacs.2c04287 (PMC9284561; doi:10.1021/jacs.2c04287)
Supplement: Supplementary file 1 — ja2c04287_si_001.pdf [file ja2c04287_si_001.pdf]

Supplementary Materials Section for

Solid-state NMR Reveals Asymmetric ATP hydrolysis in the Multidrug ABC Transporter BmrA

Denis Lacabanne[a,b,#,*], Thomas Wiegand[a,c,d,#,*], Margot Di Cesare[e], Cédric Orelle[e], Matthias Ernst[a], Jean-Michel Jault[e], Beat H. Meier[a] and Anja Böckmann[e]

[a] *Physical Chemistry, ETH Zurich, 8093 Zurich, Switzerland*

[b] *current address: Medical Research Council Mitochondrial Biology Unit, University of Cambridge, Keith Peters Building, Cambridge Biomedical Campus, Hills Road, Cambridge, CB2 0XY, United Kingdom.*

[c] *current address: Max-Planck-Institute for Chemical Energy Conversion, Stiftstr. 34-36, 45470 Mülheim an der Ruhr, Germany*

[d] *current address: Institute of Technical and Macromolecular Chemistry, RWTH Aachen University, Worringerweg 2, 52074 Aachen, Germany*

[e] *Molecular Microbiology and Structural Biochemistry, UMR5086 CNRS/University of Lyon, 7, passage du Vercors, 69367 Lyon, France*

**Corresponding authors: denis.lacabanne@mrc-mbu.cam.ac.uk, thomas.wiegand@cec.mpg.de*

[#] *Equal contributions*

Supplementary Figures Section

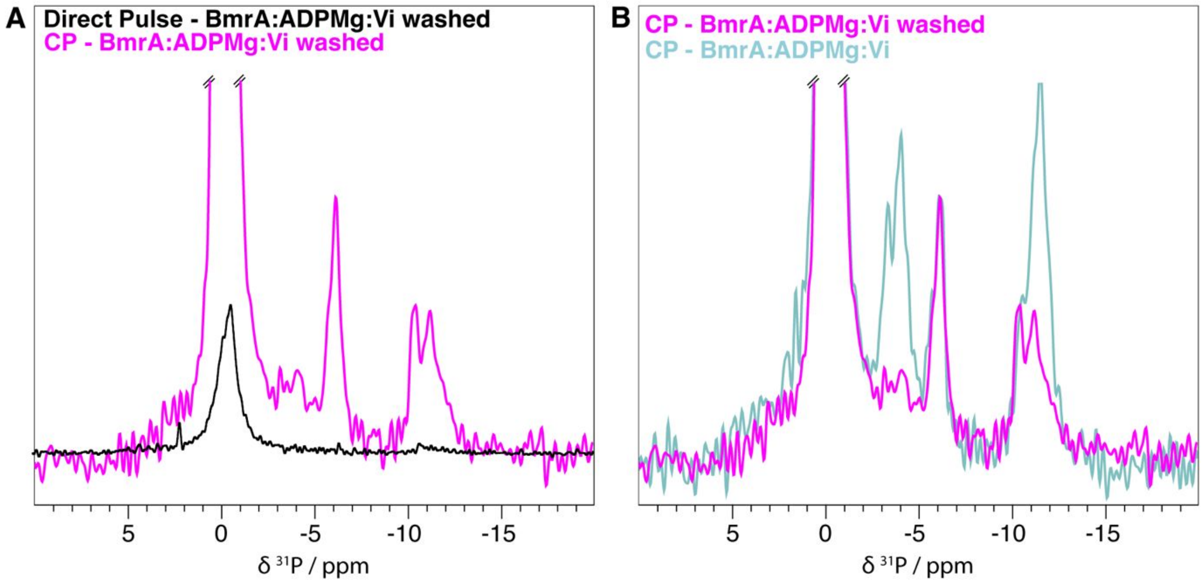

Figure S1 *^{31}P cross-polarization spectrum of BmrA:ADP:Vi.* **A.** ^{31}P CP (pink line) and direct pulsed (black line) 1D spectrum of BmrA:ADP:Vi after a washing step. **B.** Overlay of the CP spectrum of BmrA:ADP:Vi after a washing step (pink line) and of the CP spectrum of BmrA:ADP:Vi with no washing step (green line). The phosphorus peaks at -3.5 and -4 ppm are strongly attenuated when the sample is washed.

Supplementary Discussion 1

Numerical simulations of asymmetric chemical-exchange maps

Correlation peaks in the recorded 2D DARR spectra might arise from (i) spin-diffusion, (ii) chemical exchange or (iii) transferred NOE. To identify whether chemical exchange explains the observed spectral features, we have numerically calculated the 2D spectrum assuming an asymmetric three-site exchange starting from the McConnell equations[1]. We distinguish three ADP molecules occupying the chemical states 1, 2 and 3. ADP in state 1 corresponds to bound ADP which is immobilized and gives signal in cross-polarization (CP) experiments and resonates at different chemical-shift values than unbound ADP. ADP in state 2 still gives (weak) CP-signal, but the chemical-shift values are identical to the ones of ADP in solution which occupies state 3, that is unobservable by CP.

The change of ^{31}P z-magnetization during the DARR mixing period is described by the differential equation

$$\frac{d}{dt}\vec{M}_z = \mathbf{S}\vec{M}_z \qquad (1)$$

in which $\vec{M}_z$ describes the z-magnetization of ^{31}P spins of ADP molecules in the chemical states 1,2 and 3 and **S** the so-called spectral matrix, which is defined as

$$\mathbf{S} = \mathbf{R} + \mathbf{K}. \qquad (2)$$

S is a 3x3 matrix composed of the relaxation matrix comprising longitudinal relaxation (**R**) and the kinetic matrix (**K**) describing the chemical-exchange processes. Note, that any exchange process during the cross-polarization period is neglected. In case of the discussed three-site exchange, the kinetic matrix is given as

$$\mathbf{K} = \begin{pmatrix} -k_{12} - k_{13} & k_{21} & k_{31} \\ k_{12} & -k_{21} - k_{23} & k_{32} \\ k_{13} & k_{23} & -k_{31} - k_{32} \end{pmatrix}. \quad (3)$$

and the relaxation matrix is given by

$$\mathbf{R} = \begin{pmatrix} -R_1^{(1)} & 0 & 0 \\ 0 & -R_1^{(2)} & 0 \\ 0 & 0 & -R_1^{(3)} \end{pmatrix}. \quad (4)$$

where we have neglected cross-relaxation processes.

The k_{ij} are kinetic rate constants for the processes shown in **Figure S2**. The rate constants are related to thermodynamic equilibrium constants by

$$K_{12} = \frac{k_{12}}{k_{21}} \qquad (4)$$

$$K_{23} = \frac{k_{23}}{k_{32}} \qquad (5)$$

$$K_{13} = \frac{k_{13}}{k_{31}} = K_{12}K_{23}. \quad (6)$$

We now only focus on the kinetic matrix and replace **S** by **K** assuming that we can neglect longitudinal relaxation during the mixing time. The solution of the differential equation (1) after a mixing time τ is given by

$$\vec{M}_z(\tau) = \exp(\mathbf{K}\tau)\vec{M}_z(0). \qquad (7)$$

The signal intensity of the diagonal or cross peak between resonances k and l in a 2D spectrum (with k,l = 1, 2, 3), $s_{kl}(\tau)$, can be calculated from Eq. (7) (for more details see reference [2]):

$$s_{kl}(\tau) = [\exp(\mathbf{K}\tau)]_{kl}[M_z(0)]_l. \qquad (8)$$

We have calculated the signal intensities under an asymmetric exchange condition arbitrarily assuming K_{12}=K_{23}=2 and the corresponding equilibrium magnetization $\vec{M}_z(0) = \vec{M}_{z,0}$ = (1,2,4). **Figure S2B** shows a 2D spectrum-like visualisation of the resulting matrix elements. The spectrum is still symmetric, although e.g. for spins 1 more intense off-diagonal elements than diagonal peak were observed as for example also described in T_2-T_2 exchange experiments[3]. Experimentally, the ^{31}P-^{31}P DARR spectra are recorded with an initial cross-polarization step and therefore, ADP molecules in the supernatant remain unobservable. This has been considered in the simulations by assuming the non-equilibrium magnetization $\vec{M}_z(0)$ = (10,1,0). Note, that also the initial magnetization for sites 1 and 2 is not in equilibrium, since the CP-efficiencies are different as seen in the experimental spectrum (Figure 1A). The simulated spectrum assuming different chemical-shift values for sites 2 and 3 is given in **Figure S2C** and the spectrum with the same chemical shifts for sites 2 and 3 (the experimental scenario, see Figure 1B) is given in **Figure S2D**. Indeed, the simulated spectra show the same feature of an asymmetric pattern as observed experimentally.

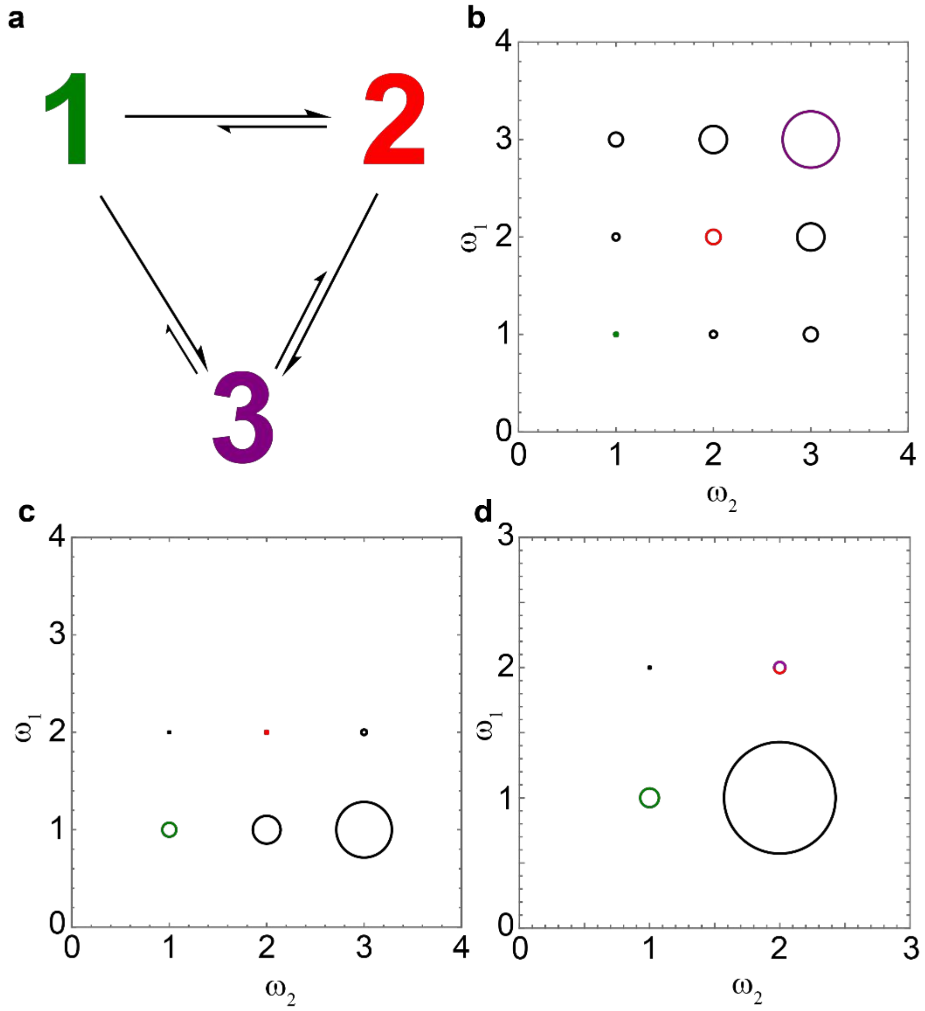

Figure S2: Three-site exchange based on the scenario sketched in **a** and simulated exchange map assuming K_{12}=K_{23}=2, τ=1 and the equilibrium magnetization $\vec{M}_z(0) = \vec{M}_{z,0}$=(1,2,4) (**b**) or K_{12}=K_{23}=2, τ=1 and the non-equilibrium magnetization $\vec{M}_z(0)$=(10,1,0) (**c**). The diagonal peaks are colour coded as in **a** and the circle size represents the signal intensity. **d** shows the simulated spectrum assuming the same exchange scenario than in **c**, but identical chemical shifts for sites 2 and 3 as observed experimentally have been chosen.

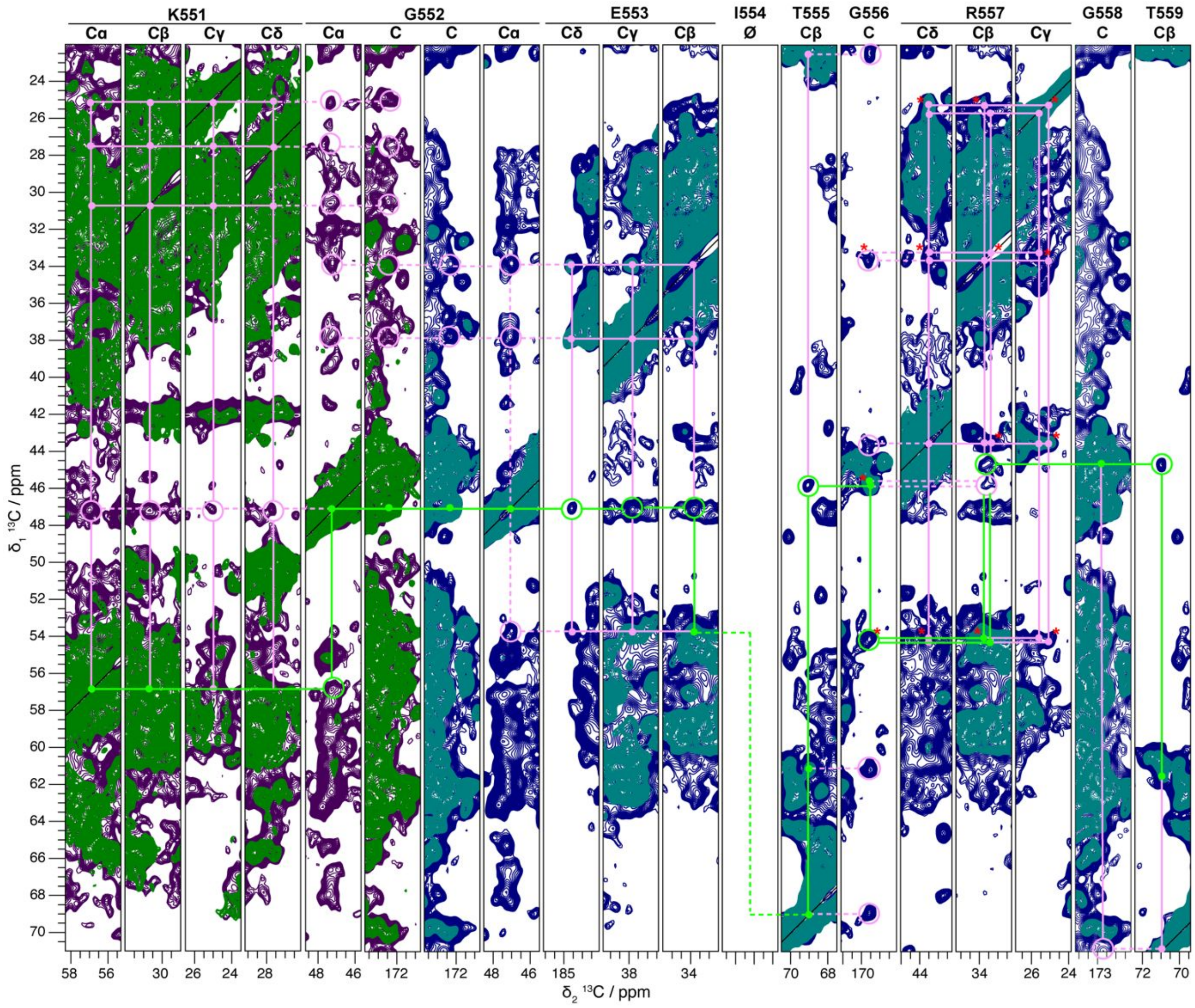

Figure S3: *Assignment pathway from residue K551 to T559.* 20 ms and 200 ms DARR spectra from sample ^{12}C-LVIRH are shown in green and dark purple respectively, while those from the sample ^{12}C-LVIKHP are plotted in teal and dark blue, respectively. The primary attribution path is shown in neon green, solid circles and lines represent intra-residue correlations, whereas empty circles and dashed lines represent inter-residue correlations (residues n-1 and n+1). The double lines are due to the presence of a peak doubling. The secondary attribution path (such as side-chains) is shown in pink. These residues were chosen as representative examples to illustrate the successful transfer of resonance assignments from solution to the solid state and to further resolve some ambiguities (for example here, the very close proximity of Cα-Cβ correlations from E553 and R557) and to highlight the presence of a peak doubling (R557) or a chemical-shift perturbation (E553) (**Figure S4**).

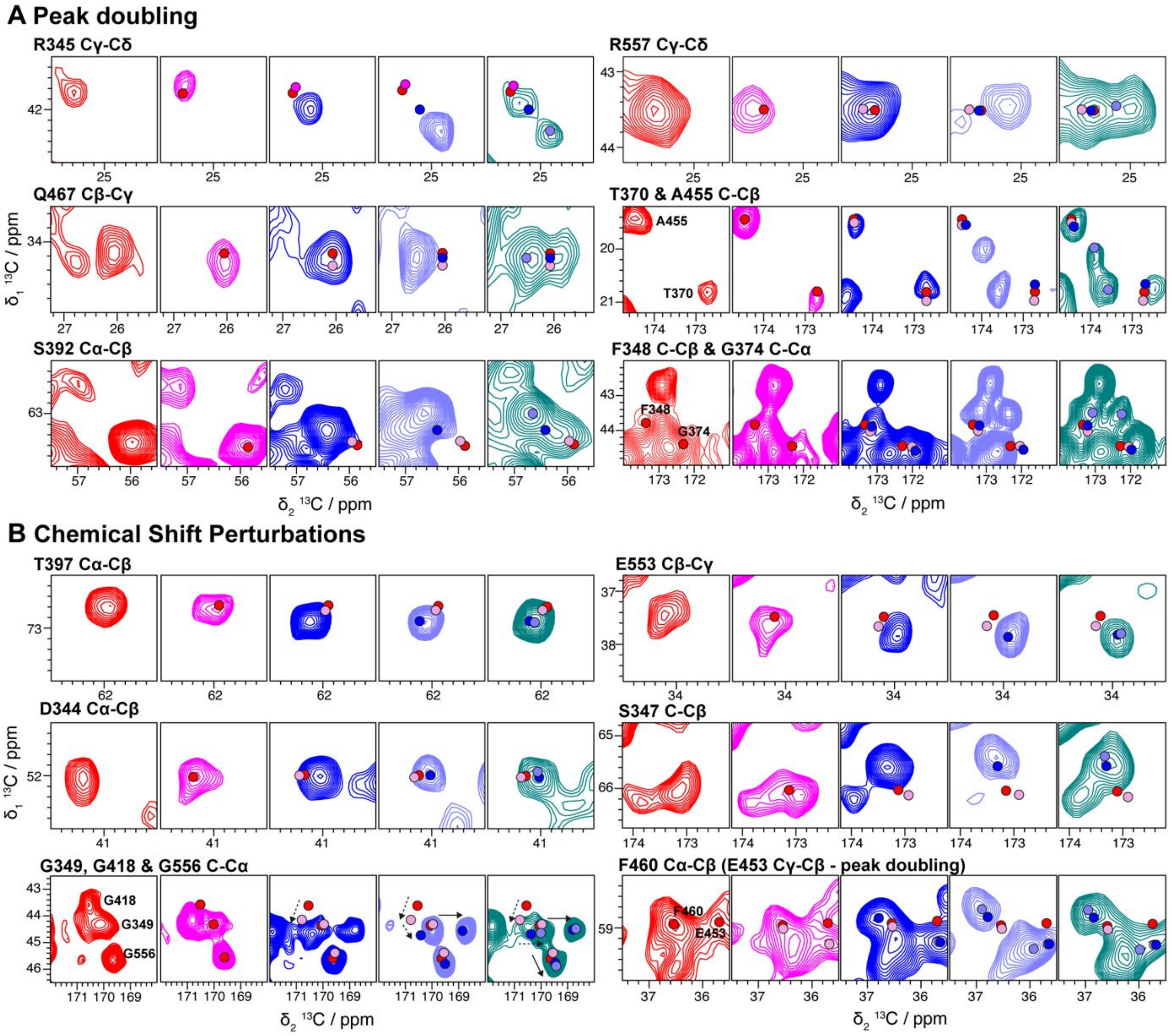

Figure S4 *BmrA conformation probed by ^{13}C-detected 2D DARR experiments*. Examples of **A** peak splittings and **B** chemical shift perturbation from WT:ADP:Vi (teal) compared with WT:ADP (dark blue), E504A:ATP (light blue) and the apo forms WT (red) and E504A (pink). Resonance assignments were transferred from solution-state NMR assignments obtained on the isolated NBD[4]. Figures of BmrA, BmrA:ADP:Vi and BmrA-E504A:ATP spectra were adapted from data previously recorded (Lacabanne *et al*. 2019[5] - Copyright 2019 Lacabanne http://creativecommons.org/licenses/by/4.0/)

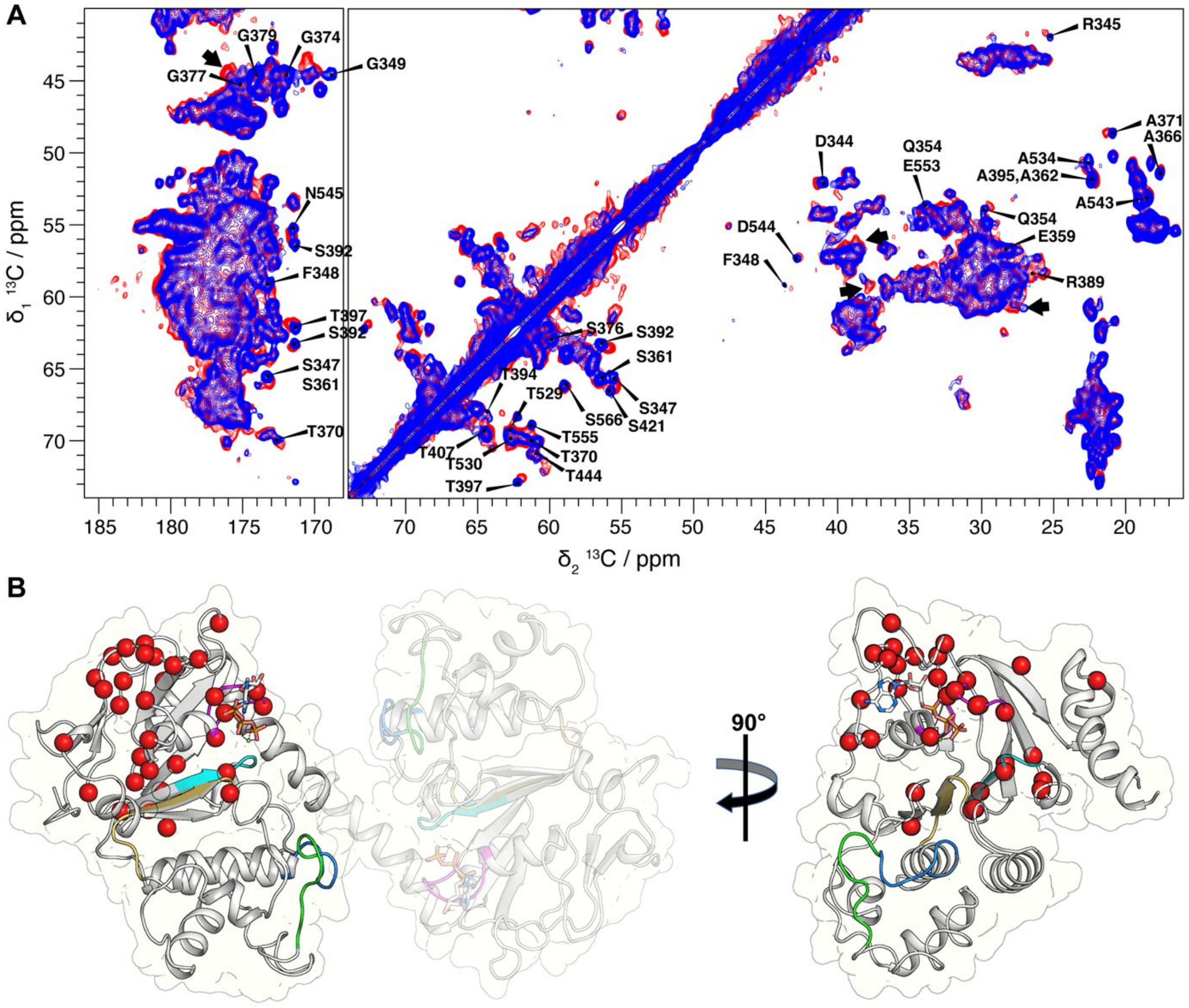

Figure S5 *Conformational changes probed by ^{13}C-detected 2D DARR experiments*. **A** Overlay of the ^{13}C-^{13}C 20 ms DARR spectra of BmrA-apo (red) and BmrA-ADP (blue). Assignment of major chemical shift perturbations are highlighted by the amino-acids number or by a black arrow when the assignment is ambiguous. **B** View of the NBDs of BmrA-E504A:ATP in the full-length structure (pdb 7OW8); the different motifs are highlighted in different colors, the X-loop (470-TEVGERG-476) in green, the Walker A motif (374-GPSGGKT-381) in magenta, the Walker B motif (496-ILMLDE-504) in dark yellow, the ABC signature (477-LSGGQ-483, in blue) and the H-loop (532-AHR-536) in cyan. All residues displaying chemical-shift perturbations determined between BmrA-apo and BmrA:ADP are shown as red spheres. Figure of BmrA spectrum were adapted from data previously recorded (Lacabanne *et al*. 2019[5] - Copyright 2019 Lacabanne http://creativecommons.org/licenses/by/4.0/)

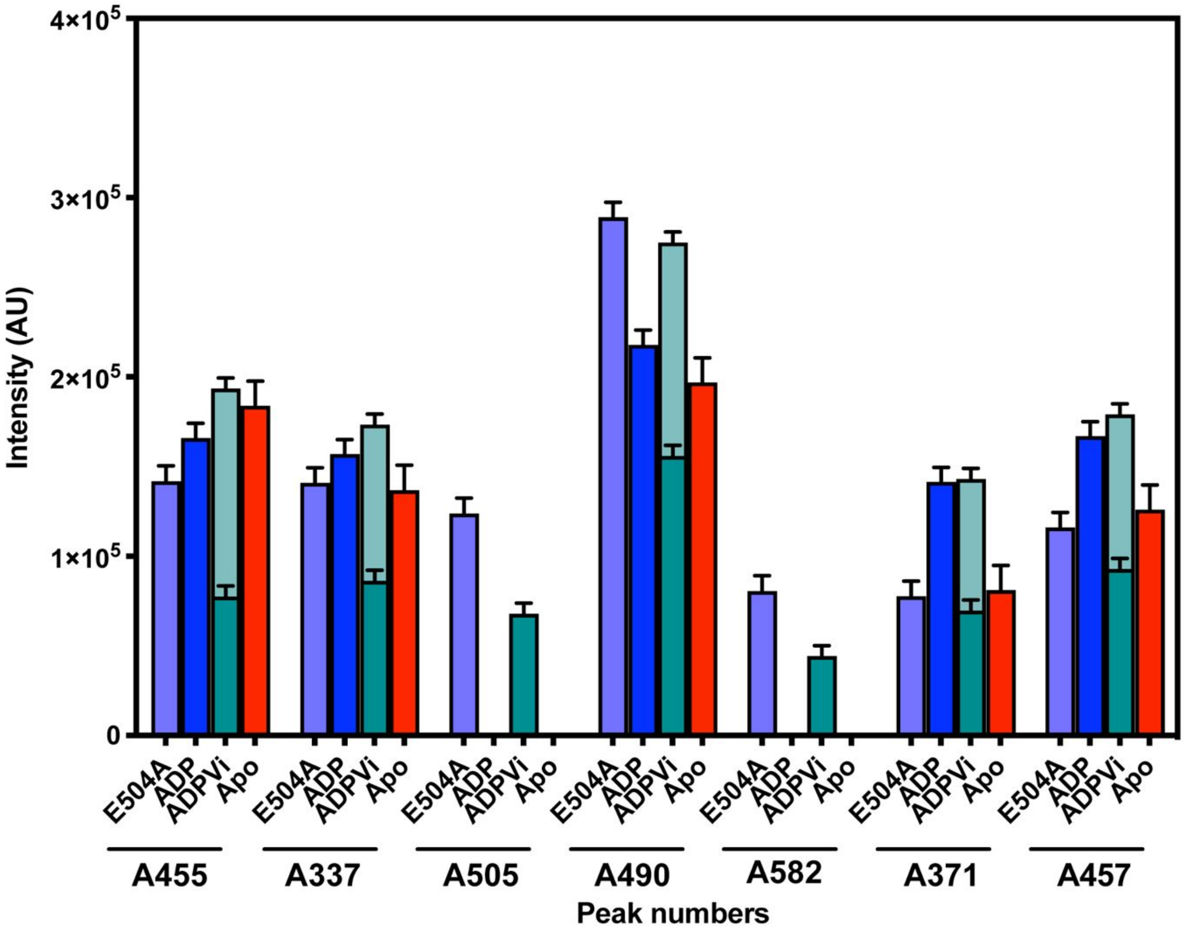

Figure S6 *Intensities of different peaks from Figure 7*. The intensities from E504:ATP (light purple), BmrA:ADP (blue), BmrA:ADP:Vi (teal) and apo (red) are plotted in a histogram for new appearing peaks (peaks 4 and 8) and peaks displaying peak-doubling for BmrA:ADP:Vi (peaks 1, 2, 7, 9 and 10). In the case of the peak doubling, the intensities of each peak were measured and presented in the same bar for BmrA:ADP:Vi.

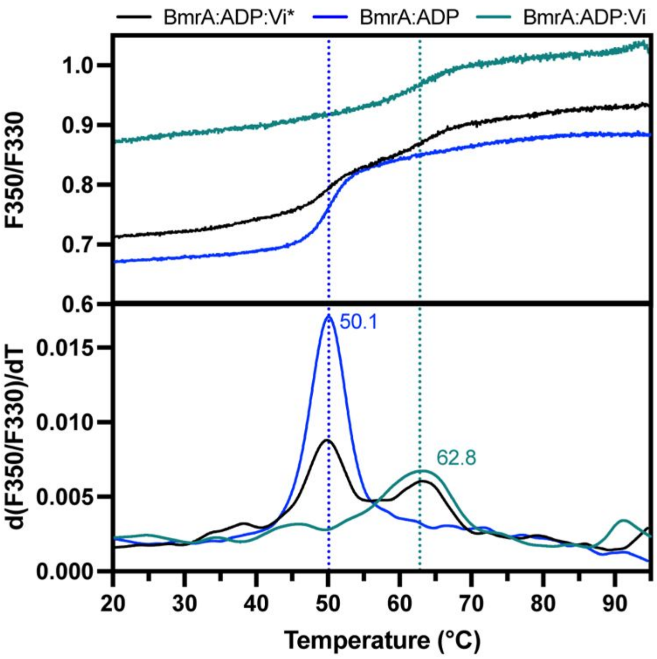

Figure S7 *Thermostability of BmrA probed by nanoDSF measurements.* Unfolding curves (top panel) and derivatives of these curves with the apparent melting temperatures (bottom panel) of BmrA in the presence of ADP and 1 mM vanadate (marked with a star), BmrA:ADP (blue line) and BmrA in the presence of ATP with 1 mM vanadate leading to the complex BmrA:ADP:Vi (green line).

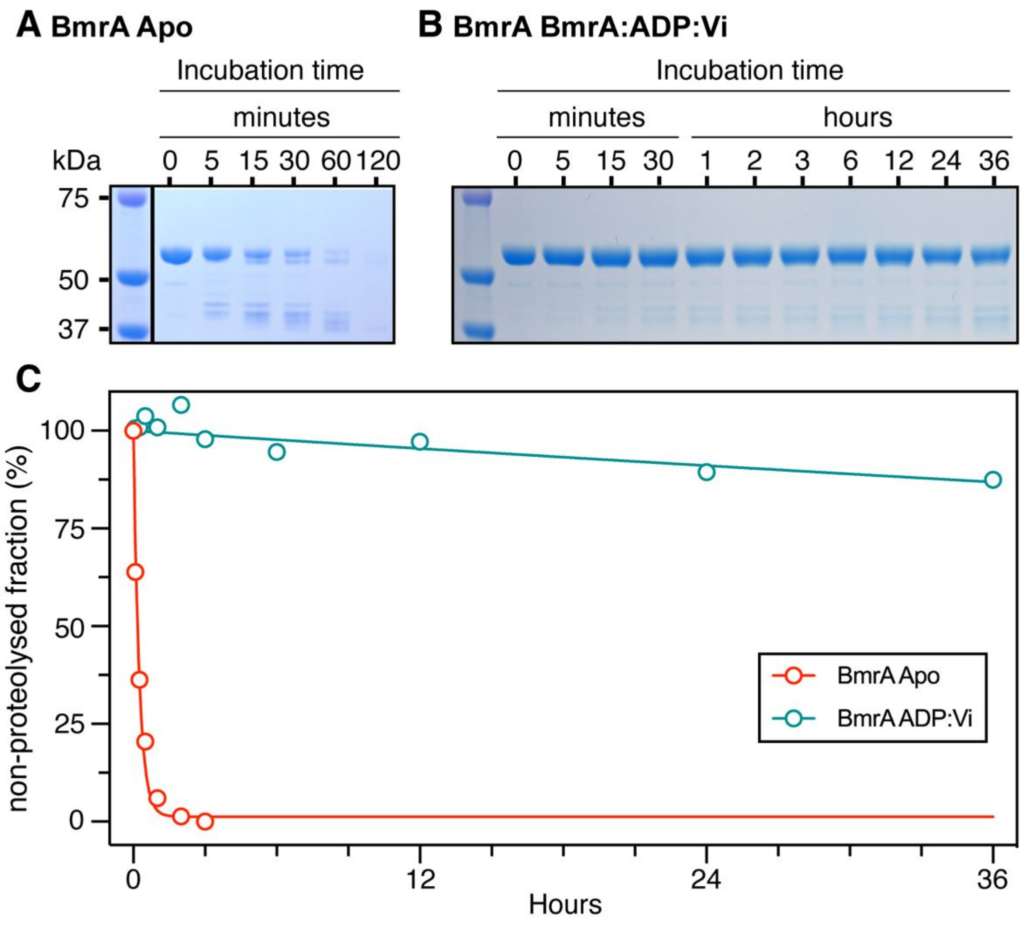

Figure S8 *Proteolysis assay.* Proteolysis assay SDS-PAGE and band quantification analysis of BmrA Apo (**A**) and BmrA:ADP:Vi (**B**). SDS-PAGE are adapted from Lacabanne *et al.* 2019[5]-Copyright 2019 Lacabanne http://creativecommons.org/licenses/by/4.0/. Band intensities were quantified by ImageJ (**C**).

Supplementary References

(1) McConnell, H. M. Reaction Rates by Nuclear Magnetic Resonance. *The Journal of Chemical Physics* **1958**, *28* (3), 430-431. DOI: 10.1063/1.1744152.

(2) Jeener, J.; Meier, B. H.; Bachmann, P.; Ernst, R. R. Investigation of exchange processes by two-dimensional NMR spectroscopy. *The Journal of Chemical Physics* **1979**, *71* (11), 4546-4553. DOI: 10.1063/1.438208.

(3) Gao, Y.; Blümich, B. Analysis of three-site T2-T2 exchange NMR. *Journal of Magnetic Resonance* **2020**, *315*, 106740. DOI: https://doi.org/10.1016/j.jmr.2020.106740.

(4) Pérez Carrillo, V. H.; Rose-Sperling, D.; Tran, M. A.; Wiedemann, C.; Hellmich, U. A. Backbone NMR assignment of the nucleotide binding domain of the Bacillus subtilis ABC multidrug transporter BmrA in the post-hydrolysis state. *Biomol NMR Assign* **2022**, *16* (1), 81-86. DOI: 10.1007/s12104-021-10063-2 From NLM.

(5) Lacabanne, D.; Orelle, C.; Lecoq, L.; Kunert, B.; Chuilon, C.; Wiegand, T.; Ravaud, S.; Jault, J.-M.; Meier, B. H.; Böckmann, A. Flexible-to-rigid transition is central for substrate transport in the ABC transporter BmrA from Bacillus subtilis. *Communications biology* **2019**, *2* (1), 149-149, 10.1038/s42003-019-0390-x. DOI: papers3://publication/doi/10.1038/s42003-019-0390-x.
